# Analysis of Multiplicity of Hypoxia-Inducible Factors in the Evolution of *Triplophysa* Fish (Osteichthyes: Nemacheilinae) Reveals Hypoxic Environments Adaptation to Tibetan Plateau

**DOI:** 10.3389/fgene.2020.00433

**Published:** 2020-05-12

**Authors:** Juan Chen, Yanjun Shen, Jing Wang, Gang Ouyang, Jingliang Kang, Wenqi Lv, Liandong Yang, Shunping He

**Affiliations:** ^1^The Key Laboratory of Aquatic Biodiversity and Conservation of Chinese Academy of Sciences, Institute of Hydrobiology, Chinese Academy of Sciences, Wuhan, China; ^2^University of Chinese Academy of Sciences, Beijing, China; ^3^State Key Laboratory of Freshwater Ecology and Biotechnology, Institute of Hydrobiology, Chinese Academy of Sciences, Wuhan, China; ^4^Center for Excellence in Animal Evolution and Genetics, Chinese Academy of Sciences, Kunming, China

**Keywords:** HIF-α, pVHL, the Tibetan Plateau, hypoxia adaption, *Triplophysa* fish

## Abstract

HIF (Hypoxia-inducible factor) gene family members function as master regulators of cellular and systemic oxygen homeostasis during changes in oxygen availability. Qinghai-Tibet Plateau is a natural laboratory for for long-term hypoxia and cold adaptation. In this context, *T. scleroptera* that is restricted to >3500 m high-altitude freshwater rivers was selected as the model to compare with a representative species from the plain, *P. dabryanus*. We cloned different HIF-α and carried out a phylogenetic analysis from invertebrates to vertebrates for identifying HIF-α genes and analyzing their evolutionary history. Intriguingly, the HIF-α has undergone gene duplications might be due to whole-genome duplication (WGD) events during evolution. PAML analysis indicated that HIF-1αA was subjected to positive selection acted on specific sites in *Triplophysa* lineages. To investigate the relationship between hypoxia adaptation and the regulation of HIF-α stability by pVHL in plateau and plain fish, a series of experiments were carried out. Comparison the luciferase transcriptional activity and protein levels of HIF-αs and the differing interactions of HIF-αs with pVHL, show clear differences between plateau and plain fish. *T. scleroptera* pVHL could enhance HIF-α transcriptional activity under hypoxia, and functional validation through pVHL protein mutagenesis showed that these mutations increased the stability of HIF-α and its hetero dimerization affinity to ARNT. Our research shows that missense mutations of pVHL induced evolutionary molecular adaptation in *Triplophysa* fishes living in high altitude hypoxic environments.

## Introduction

Tibetan Plateau is commonly referred to as the “Roof of the World” (Zhou et al., 2006), which is the highest and largest plateau on earth. Its inhospitable environment, characterized by severe coldness, severe hypoxia and strong ultraviolet radiation (Su and Wang, 1992; Bickler and Buck, 2007; Cheviron and Brumfield, 2012), has profound effects on animal survival and thus, it is regarded as a global biodiversity hotspot and a natural laboratory for long-term hypoxia and cold adaptation studies research (Wu and Wu, 1992; Scheinfeldt and Tishkoff, 2010; Wang et al., 2011; Qi et al., 2012; Zhang et al., 2017). The oxygen content varies markedly daily, seasonally, and spatially (Soitamo et al., 2001). The dissolved oxygen in water decreases with increasing altitude, for example at 4500 m elevation, the oxygen content in the water is only 2 mg/l (Xu et al., 2016). As a representative of the endemic fishes of the Qinghai-Tibet Plateau, *Triplophysa scleroptera* (Cypriniformes: Balitoridae: Nemacheilinae) (Herzenstein, 1888) has become an interesting model for studies adapted to the cold and hypoxic conditions of its high-altitude habitat. In contrast, *Paramisgurnus dabryanus* is widely distributed in the middle and lower reaches of the Yangtze River Basin. The special geographical distributions provide a rich source of naturally occurring genetic variation between species, which can be used to explore the molecular mechanism of hypoxia adaptation. Previous researches primarily used high-throughput sequencing data (e.g., mitochondrial genome and transcriptome) to explore the genetic mechanisms of high altitude adaptations (Yang et al., 2014; Wang et al., 2015a, b, 2016). However, no study has been made so far regarding the HIF pathway in Plateau fish; therefore, the impact of transcriptional factors and/or their target genes on the mechanisms of hypoxia and post-hypoxia adaptation remains elusive. In this study, we focused on the underlying molecular mechanism of hypoxia adaptation to discover the potential genetic and physiological mechanism of its adaptation to high-altitude environments.

The hypoxic signaling pathway is a system of cellular signaling pathways that is very conserved from nematodes to mammals. The maintenance of oxygen homeostasis is vital to the survival of organisms, which requires coordinated regulation of various genes. Hypoxia-inducible factor is the most critical factor in this signaling system, and recent studies have shown regulation and function of HIF-α subunits in fish inhabiting different hypoxic environments (Rahman and Thomas, 2007; Terova et al., 2008; Guan et al., 2014). HIF consists of two subunits: HIF-α and HIF-β (also known as the aryl hydrocarbon receptor nuclear translocator, ARNT), which belongs to the basic helix-loop-helix-PER-ARNT-SIM (bHLH-PAS) family. It’s the major activator of downstream genes, which played a central role in the development, physiology, pathology and the adaptive response to hypoxia (Wang et al., 1995; Semenza, 1999; Bickler and Buck, 2007). HIF-α is tightly regulated by oxygen levels, whereas HIF-β is stable (Semenza, 2012). Like other vertebrates, there are multiple forms of HIF-αs in fish. Moreover, cyprinids have duplicate copies of all three HIF-α genes, a possible of an ancient teleost-specific third round of genome duplication (Meyer and Van de Peer, 2005; Opazo et al., 2013; Rytkonen et al., 2013; Guan et al., 2014). A key regulator of HIF-α is von Hippel Lindau (pVHL) tumor suppressor protein (Tarade et al., 2019),which has two domains called α and β(Stebbins et al., 1999; Cockman et al., 2000). Under normal oxygen conditions, pVHL inhibits HIF-α activity by targeting the HIF-α subunits for polyubiquitination (Epstein et al., 2001; Jaakkola et al., 2001). Under hypoxic conditions, hypoxia reduces prolyl hydroxylase enzyme (PHD1-3) activity, stabilizing HIF-α, which joins a nuclear complex with the constitutively expressed HIF-β and transduces the cellular response by binding to hypoxia-responsive elements (HREs; A/GCGTG) in the promoter regions of its target genes to activate gene transcription (Wang and Semenza, 1993; Wang et al., 1995; Schofield and Ratcliffe, 2004; Kaelin and Ratcliffe, 2008; Webb et al., 2009).

The relationship between HIF-αs and pVHL is considered one of the main factors that influence hypoxia signaling pathways (Bi et al., 2017). Defining the mechanisms of HIF-α regulation by pVHL will increase our understanding of the hypoxia signaling pathway and the mechanisms of hypoxic adaptation and tolerance in highland fish. To date, the genetic mechanisms of adaptations to hypoxia have been extensively studied in the Tibetan people (Beall et al., 2010; Xu et al., 2011; Bigham and Lee, 2014), Tibetan mastiff (Gou et al., 2014), Tibetan gray wolf (Zhang et al., 2014), cave mammals (Shams et al., 2004), ectothermic snakes (Li et al., 2018), and diving cetaceans (Tian et al., 2016; Bi et al., 2017). However, compared to mammals, only limited amounts of genetic or other information are available on the adaptations of fishes. Thus far, the study on the plateau adaptability of fishes is mainly based on the transcriptome and mitochondrial genome, investigations into changes in evolutionary rates and to identification of the potential genetic bases for high-altitude adaptations (Ma et al., 2015; Wang et al., 2015a, b, 2016). Tarade et al. explored evolution of metazoan oxygen-sensing and found the effect of pVHL on HIF-α was different (Tarade et al., 2019). However, the underlying molecular mechanisms of hypoxia adaptation in the plateau loach remain unknown, and the important regulatory factors in the hypoxic pathway, including HIF-α and pVHL, have not been studied (Burga et al., 2017). In this study, to better understand the relationship between the adaptation to hypoxia and the regulation of HIF-α stability by pVHL in plateau and plain fish, we cloned and characterized four distinct HIF-α isoforms (HIF-1αA/B and HIF-2αA/B) and pVHL genes to determined their expression levels and impact on physiological regulation in *T. scleroptera* (highland loach) and *P. dabryanus* (plain loach), attempted to find differences in the hypoxic signaling pathways between plateau fish and plain fish. This findings substantially advanced our understanding of evolutionary biology and functional adaptation to the hypoxic ecological environment of the plateau.

## Materials and Methods

### Sample Collection

Experiments involving animals in this study were conducted in accordance with the Laboratory Animal Management Principles of China. All procedures involving the use of fish were approved by the ethical board of the Animal Care and Use Committee of the Institute of Hydrobiology, Chinese Academy of Sciences (protocol number IHB2017001). An adult male *T. scleroptera* (YC) was collected in upper reaches of the Yellow River (3600 m in elevation), where the low oxygen tension exerts unique selection pressures. Tissue samples from five major organs including heart, liver, brain, spleen, and kidney were collected immediately and placed in liquid nitrogen during field and transportation, subsequently transferred to the −80°C freezer for long term storage. An adult male *P. dabryanus* (PD) was collected in middle reaches of the Yangtze River Basin in Wuhan of Hubei Province (about sea level), and tissues including heart, liver, brain, spleen, and kidney were also collected in the same way. All of the instruments were treated with DEPC water.

### RNA Extraction, and cDNA Preparation

Total RNA was, respectively, extracted from tissue samples (heart, liver, brain, spleen, and kidney) using the TRIzol reagent (Invitrogen, Karlsruhe, Germany), following the manufacturer’s instructions. RNA degradation and contamination was monitored using 1% agarose gels. RNA concentration and the quality were checked by spectrophotometry (optical density 260/280 ratio) using a NanoDrop 2000 (Thermo Fisher Scientific, United States). RNA samples with OD260/280 ratios between 1.8 and 2.1 were treated with DNase I (DNA-free, Ambion). Equal quantities of RNA were pooled from each tissue to create one mixed sample. We used 2 μg of total RNA as a template to synthesize the first strand cDNA using reverse transcriptase (Promega) in the presence of RNase inhibitor (Invitrogen) with a cocktail of oligoT and dNTP (TaKaRa) and rapid amplification of cDNA ends (RACE) using a Clontech SMARTer^TM^ RACE cDNA Amplification Kit. Aliquots of 1 ul of undiluted cDNA were used per PCR.

### Molecular Cloning and Plasmid Constructs

We searched for these desired genes (HIF-αs and pVHL) in the available transcriptome sequences. The genome-wide survey was based on TBLASTN searches in the separate transcriptome assemblies: *T.scleroptera* (PRJNA280009), and *P. dabryanus* (PRJNA266739). ORF finder on NCBI was used to predict the amino acid sequences of HIF-αs and pVHL. SMART (Letunic et al., 2015) and UniProt^1^ were utilized to check protein domains. Based on our transcriptome data, we designed a pair of primers by using Primer Version 5.00 (Primer Biosoft International, Palo Alto, CA, United States), to obtain the true sequence of the gene by PCR technology. Primers for amplifying are listed in Additional file 1: Table S1. Subsequently, the PCR products were recombined with pMD18-T vector (TaKaRa), sequenced using an ABI3730XL sequencer, and assembled using SeqMan in DNASTAR Lasergene v7.1 software (DNASTAR Inc.). Mutagenesis of the pVHL sequence (P92 T, S109 T, and P92 T+S109 T) was achieved through site-directed mutagenesis and verified by Sanger sequencing. Briefly, the complementary oligo deoxyribo-nucleotide primers were designed at the mutation sites, and standard PCR cloning techniques were used to generate fragments that included the mutations. The correct and intact full-length pVHL, HIF-1αA/B and HIF-2αA/B from *T. scleroptera* and *P. dabryanus* were isolated from one of the TA clones using newly designed gene-specific primers to the 5′ and 3′ ends with restriction enzyme sites (Additional file 2: Supplementary Table S2), and ligated into the expression vector PCMV-HA (CloneTech), and the PCMV-Myc vector (CloneTech). All recombined expression plasmids were verified by Wuhan Tianyi Huiyuan Bioscience & Technology Corporation (Wuhan, China).

### Sequence and Evolutionary Analysis

For phylogenic analysis, sequences of the HIF-α (HIF-1αA/B, HIF-2αA/B) and pVHL were obtained from GenBank^2^ and Ensemble^3^. In total, 70 homologous sequences for HIF-α were retrieved and sequence identity was performed using online tools. Sequence alignments were performed using MUSCLE (Edgar, 2004). Additionally, jModeltest 2.1.10 (Posada, 2008) was used to choose the best models for nucleotide substitution. Finally, a phylogenetic tree was constructed by MrBayes-3.2.2 (Huelsenbeck and Ronquist, 2001) with 20,000,000 iterations. To investigate whether HIF-α and pVHL genes have undergone statistically significant differences in selection pressures, we employed branch-site model in the CodeML program (PAML V4.8) (Yang, 2007) to detect the positive selection that may have affected specific sites along a specific branch. In the branch-site model (model = 2, Nsites = 2); the neutral model, which constrains a class of sites to ω = 1(fix omega = 1, omega = 1); and the selection model that allows a class of codons in the foreground branch to have ω > 1 (fix omega = 0, omega = 1.5). Likelihood ratio tests were used to compare these nested models with a χ2 distribution was used to identify positively selected codons. Positive selected sites were detected by the Bayes empirical Bayes method (Yang et al., 2005). In addition, the PROVEAN (Choi et al., 2012) was used to predict whether an amino acid substitution or indel has an impact on the biological function of a specific site. Variants most likely alter protein function. The PROVEAN score was calculated for each variant; the more negative the score, the more likely a given variant alters the protein function. Furthermore, the 3D structures of the pVHL gene were constructed by using Swiss-Model (Bordoli et al., 2009), and the subsequent pVHL molecular visualization performed by using PyMOL (Schrodinger, 2010).

### Cell Culture

Human embryonic kidney (HEK) 293 T cells were used because they possess high transfection efficiency. HEK 293 T cells were cultured in Dulbecco’s modified Eagle’s medium (DMEM, Hyclone) supplemented with 10% fetal bovine serum (FBS, Hyclone) and 1% penicillin and streptomycin at 37°C in a 5% CO2 humidified incubators (Thermo). Hypoxia treatment was conducted in a hypoxic chamber with 2% oxygen concentration by using an incubator with O2 control filled with 5% CO2 and balanced with N2(NBS Galaxy 48R). Cells were plated in six-well plates or twenty four -well plates at 50–70% confluence 1 day before the transient transfection.

### Western Blot Analysis

For protein expression assays, HEK 293 T cells were seeded in 6-well plates for 12 h before transfection, and then transfected with the indicated amounts of plasmids using VigoFect (Vigorous) reagent. Transfected cells were incubated in fresh medium and divided into two groups and cultured in normoxic or hypoxic chambers (2% O2) (Ruskinn INVIVO2 I-400) for 18 h. After two consecutive washing steps with PBS, the cells were harvested in RIPA buffer (Beyotime) with 1% PMSF (Beyotime), 1% Na_3_VO_4_, and a phosphatase inhibitor cocktail (Thermo Scientific) was added. All operations are done on ice for 50 min, and cell debris was removed by centrifugation at 13,000 rpm for 15 min. Then the lysate was mixed with 5× protein loading buffer (Beyotime), and boiled for 10 min. The antibodies were used as follows: anti-c-Myc antibody (9E10, Santa Cruz), anti-HA antibody (Covance), and anti-GAPDH antibody (60004-1-Ig). HRP labeled goat anti-mouse IgG (H + L, Beyotime) was used as the secondary antibody. We used a Fujifilm LAS4000 luminescent image analyzer to photograph the blots. ImageJ software was used to evaluate quantify protein levels based on density of the protein bands. For the analysis of the degradation rate of HIF-αs in the highland loach and plain fish, Myc-tagged HIF-αs was transfected into the HEK293T cells in the presence of cycloheximide (CHX, 10 μg/ml; Sigma, Munich, Germany), which was used to block the synthesis of endogenous HIF-αs.

### Transfections and Luciferase Reporter Assays

Cells were seeded in 24-well plates for 12 h under normoxia (21% O2) before transfection and then were transfected with the indicated amounts of plasmids using VigoFect (Vigorous) together with the hypoxia response element luciferase reporter (HRE-Luc.) following the instructions recommended in the manufacturer’s protocol. pRL-Renilla was used as an internal control. Luciferase activity was measured 24 h after transfection using the Dual-luciferase Reporter Assay System (Promega). Luciferase activity data was normalized to *Renilla* luciferase. All experiments were performed in three independent operations.

### Statistical Analysis

Luciferase assays data were normalized to Renilla luciferase and presented as mean ± SE.M. of three independent experiments performed in triplicate. We compared mean relative luciferase activity by using unpaired Student’s *t* tests in GraphPad Prism 5 (GraphPad Software, La Jolla, CA, United States).

## Results

### Characterization of HIF-α Duplications and pVHL Gene

**FIGURE 1:**
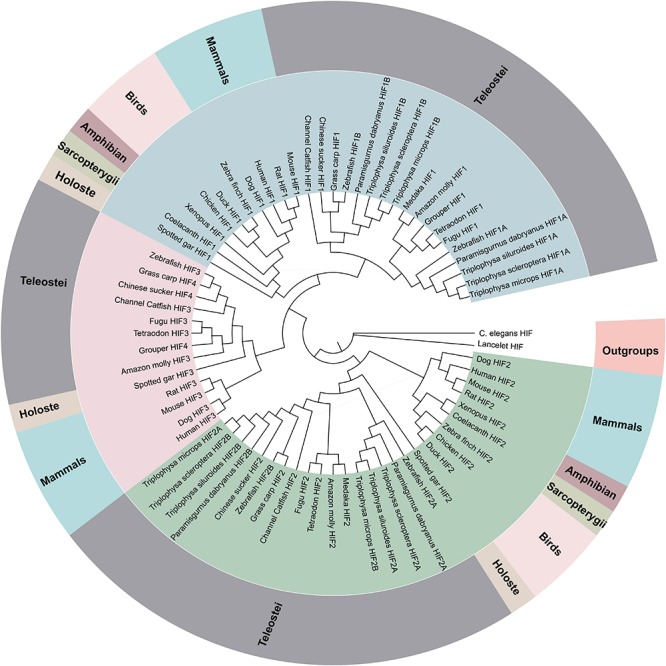
Phylogenetic tree of the HIF-α genes. The maximum likelihood method was used to reconstruct the tree that was rooted with invertebrate HIF-α sequences. Species are listed along with their phylums or classes.

The sequences of the HIF-α genes (HIF-1αA/B and HIF-2αA/B) and pVHL were obtained here for high -land *Triplophysa* fish and for fishes living at low altitudes (*P. dabryanus*). Bioinformatics analysis showed that all HIF-α/pVHL genes contained complete ORF regions, and the biochemical and physical parameters are listed in Additional file 3: Supplementary Table S3. A computer analysis indicated that the *T. scleroptera* ORF of the HIF-1αA, HIF-1αB, HIF-2αA and HIF-2αB cDNA encodes for a putative protein of 686,776, 843, 817 amino acids, respectively, and shares ∼40–56% sequence similarity with the mammalian HIF-αs, and high shared identity with other *Triplophysa* fishes. On the other hand, analysis of the pVHL cDNA showed that it encodes a putative protein of 171 amino acids, which shares >80% sequence similarity with PD pVHL (Additional file 4: Supplementary Table S4). Both HIF-1αA/B and HIF-2αA/B deduced proteins contain the characteristic domains, similar to those of their mammalian counterparts: bHLH domain, PAS-A and PAS-B domains, PAC motif, an oxygen-dependent degradation domain (ODDD), and two TADs domain (Supplementary Figures S1, S2). Furthermore, the HIF-α subunits have N-NLS and C-NLS (nuclear localization signals), respectively. HIF-1αA and HIF-1αB were about 60% identical, while HIF-2αA and HIF-2αB were 62.23% identical. HIF-1α is structurally similar to HIF-2α; these two subunits share 43% amino acid sequence identity and are mainly differed in the N-TAD domain. We found that the N-terminal domains (bHLH, PAS- A/B, and C-TAD) were highly conserved, while the C-terminal domains especially ODDD, which responsible for the oxygen-dependent degradation of the protein (Ivan et al., 2001; Rytkonen et al., 2007), had lower identity values. This suggesting that they may be differences in their sensitivity to hypoxia. The hydroxylation targets, the proline and the asparagine which are located in the ODDD, C-TAD domain, respectively, are conserved, despite the highly variable sequences among them. In addition, we found some shared amino acid replacements in the HIF-α paralogs of the Tibetan loach, which is located in important domains (Figure 1, Supplementary Figure S3). At the same time, we found that some basic amino acids that interact with DNA, namely, K19, K21, R27, R29, R30, D55, and K56 (HIF-1α), appear to be conserved across species, indicating integrity function with DNA binding (Supplementary Figures S1, S2).

Sequence analysis indicated that the deduced amino acid sequence of pVHL also included characteristic α/β domain similar to mammalian counterparts. Some amino acid sites were uniquely found in the high-altitude loach, which differed from lower-altitude pVHL (Supplementary Figure S4). It is well known that site differences often determine a difference in function. Whether the difference between these sites affects the function of the gene, should be explored. We used PROVEAN to calculate the possible impact of each variant, and the default threshold was -2.5. When the score equal to or less than -2.5 were considered “deleterious,” while the score greater than −2.5 were considered “neutral.” The more negative the score, the more likely a given variant is to alter the protein function. Most variants were predicted to be neutral; however, the proline-to-threonine replacement at position 92, which is located in the β domain (the HIF-binding domain), was predicted to be deleterious (Additional file 5: Supplementary Table S5). In addition, another amino replacement uniquely exists in YC-pVHL at position 109 in the α domain (Supplementary Figure S4B), where ElonginC binds in association with ElonginB to recruit the pVHL–ElonginB–ElonginC complex to Cul2-Rbx1, which forms a ubiquitin-protein ligase.

### Phylogenetic Analysis and Selective Pressure Analysis

The phylogenetic analysis of the HIF-αs across different organisms or species with MrBayes recovered the tree shown in Figure 1. Regarding early eukaryotic evolutionary history, only one HIF-α has been discovered in the genomes of the examined invertebrates, such as *Caenorhabditis elegans*. However, where amphioxus at the most basal of chordate subphylum has a single HIF-α gene, vertebrates often have two or more paralogs derived from two whole-genome duplication events, suggesting that amphioxus were the ancient origin of the HIF-α family. The additional two paralogs (HIF-1αA/B and HIF-2αA/B) were present in cyprinids most likely a result from the teleost-specific whole-genome duplication (Rytkonen et al., 2013) (Additional file 6: Supplementary Table S6). The topology of HIF-α members were largely consistent with the results based on mitogenome and transcriptome data in previous studies. The phylogenetic analysis strongly support three clades (Figure 1). The deduced amino acid sequences of HIF-3α and HIF-4α forming a separate group, as a sister group of HIF-1α.

To better understand whether the hypoxia-related gene was subjected to natural selection during plateau adaptation, we estimate the frequency on the coding sequences. We used the one ratio model in the CodeML for the phylogenetic analysis with maximum-likelihood (ML) software (PAML V4.7). The ω values of these hypoxia-related gene ranged from 0.15 to 0.17 and were significantly <1 (Additional file 7: Supplementary Table S7), suggesting under strong purifying selection process in the evolution.

**FIGURE 2:**
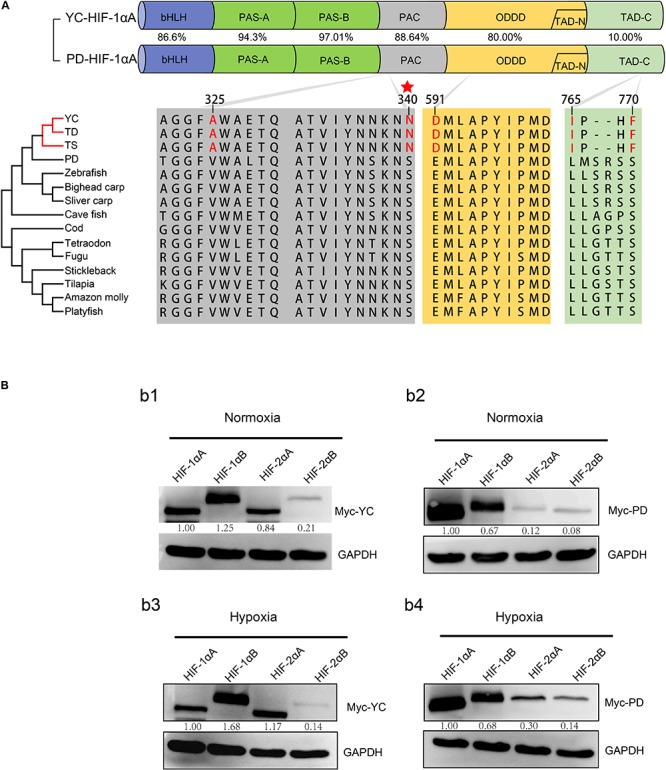
**(A)** Domain structure of hypoxia-inducible factor HIF-1αA. The following domains are shown: basic helix-loop-helix domain (bHLH), Per-Arnt-Sim homology domain (PAS-A/B), a PAS-associated COOH-terminal (PAC) motif, O2-dependent degradation domain (ODDD), NH2- and COOH-terminal transactivation domains (TAD-N and TAD-C). Percentage represents the similarity ratio of amino acid sequence identities between functional domains from highland loach (YC: *T. scleroptera*) and plain loach (PD: *P. dabryanus*). Shared amino acid replacements in *Triplophysa* fishes compared with all low-elevation species are shown in red. The red star marks the amino acid replacement experienced positive selection, which identified by branch-site model with a posterior probability greater than 0.95. TD, *T. dalaica*; TS, *T. siluroides*. **(B)** Expression of different HIF-αs in HEK 293T cells under normoxic and hypoxic conditions. (b1) The levels of HIF-α paralogs protein from the *T. scleroptera* (YC) under normoxic condition. (b2) The levels of HIF-α paralogs protein from the *P. dabryanus* (PD) under normoxic condition. (b3) The levels of HIF-α paralogs protein from the *T. scleroptera* (YC) under hypoxic condition. (b4) The levels of HIF-α paralogs protein from the *P. dabryanus* (PD) under hypoxic condition.

To investigate whether positive selection acted on specific sites in *Triplophysa* lineages, we used branch-site models in PAML. In all the HIF-α paralogs, we found that only HIF-1αA (*p* < 5.933e-07) subjected to positive selection that acted on specific sites. By using the BEB (Bayes empirical Bayes) method, there were 16 amino sites subjected to positive selection in HIF-1αA with a posterior probability greater than 0.5 (Additional file 8: Supplementary Table S8). Some of the *Triplophysa* lineage-specific nonsynonymous mutations were identified, including 325A, 340N, 591D, 765I, and 770F (Figure 2A). The 340th asparagine had a high posterior probability of 0.989, which was significant at the 5% confidence interval. The highland loaches possess a unique amino acid asparagine, in an important PAC domain; however, serine, not arginine, was found in all representative vertebrates, and may be important for PAS domain folding (Maynard and Ohh, 2004). For pVHL, the branch-site model was also carried out to detect positive selected sites along a specific branch. When we set all *Triplophysa* fishes as the foreground branches, did not find any positive selection signal. To further explore the potential difference, a clade of *T. scleroptera* was set as the foreground branches, and we found some unique, likely positive selection sites (19A, 61T, 98A, and 109S in *T. scleroptera*, which all located in important domains (Supplementary Figure S4B).

### HIF-α Paralog Proteins Have Divergent Expression

**FIGURE 3:**
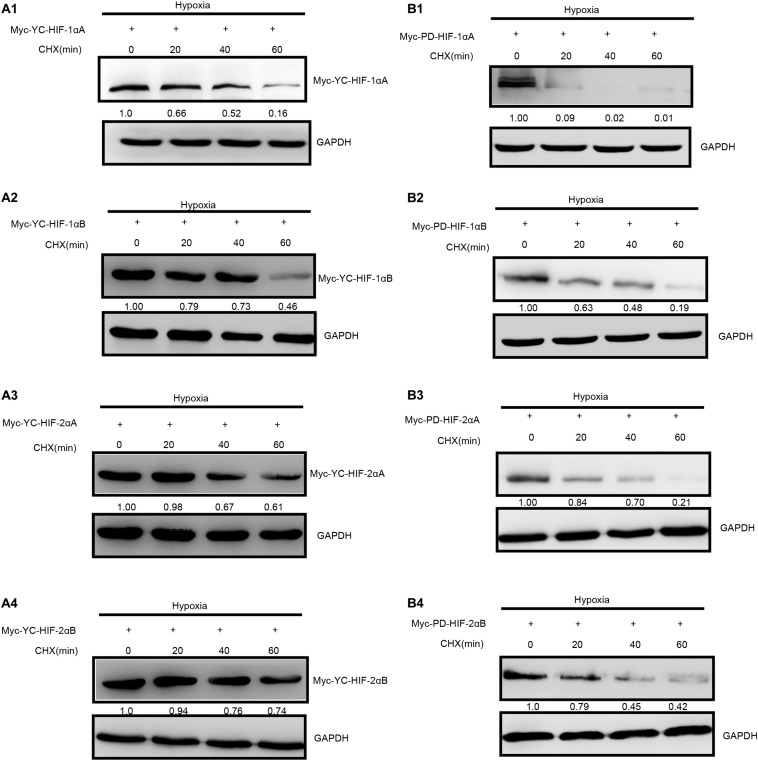
Degradation rate of *T. scleroptera* (YC) HIF-αs differs from that of the *P. dabryanus* (PD). **(A1–A4)** The protein levels of YC HIF-αs protein at different time points after exposure to hypoxia. **(B1–B4)** The protein levels of PD HIF-αs protein at different time points after exposure to hypoxia. All constructs were detected at similar levels. Cycloheximide (CHX) was added to the cells and cell lysates were prepared at the indicated time points.

As two major regulatory factors of the hypoxia signaling pathway, HIF-1α and HIF-2α have garnered long-term interest in distinguishing their roles, and related research have shown that both HIF-1α and HIF-2α participate in hypoxia-dependent gene regulation (Raval et al., 2005). The discovery of differences between HIF-α isoforms could advance our understanding of high-altitude hypoxic stress response mechanisms. To test the potential functional differences of the HIF-αs protein in highland loach compared with the lowland species (Supplementary Figure 3A), we compared of these protein level under hypoxic or normoxic conditions. Western blot analysis displayed that all the fish HIF-αs could be expressed in HEK 293T cells and the protein expression levels of each HIF-α subunits are different from each other. Compared to the other HIF-α subunits, HIF-1αB protein expression level is the highest neither hypoxic conditions nor normoxic conditions in high-elevation fish. Nevertheless, HIF-1αA is the most highly expressed isoform in plain fish (Figure 2B). Generally, whether under normoxia or hypoxic conditions, HIF-1α was greater protein expression level than HIF-2α, which implying that HIF-1α may be acted as an important regulator of fish in response to the environmental hypoxia (Figure 2B). Compared to those of highland fish, the increase in the levels of protein expression of plain fish was rare. Subsequently, to investigate whether there is a difference in hypoxia-induced regulation of HIF-1αs and HIF-2αs protein stability between highland and plain fish, CHX was used to block new protein synthesis. We found that HIF-α protein from the *P. dabryanus* (PD) were degraded at a higher rate than those from the *T. scleroptera* (YC) in response to hypoxia (Figure 3). The degradation rate of HIF-α was slower in YC, suggesting long-term highland exposure enhanced gene expression function for adaptation to hypoxic environments. These results prompted us to ponder whether other genes in the signaling pathway might invoke differences between highland fish and low-elevation fish.

### HIF-2αA More Efficiently Induced Target Gene Expression Compared With Other HIF-α Isoforms

To assess the transcriptional activity of different HIF-α paralogs in highland loach and plain loach, Myc-tagged HIF-α plasmid were cotransfected with HRE and pRL-Renilla luciferase reporters into HEK 293T cells.

**FIGURE 4:**
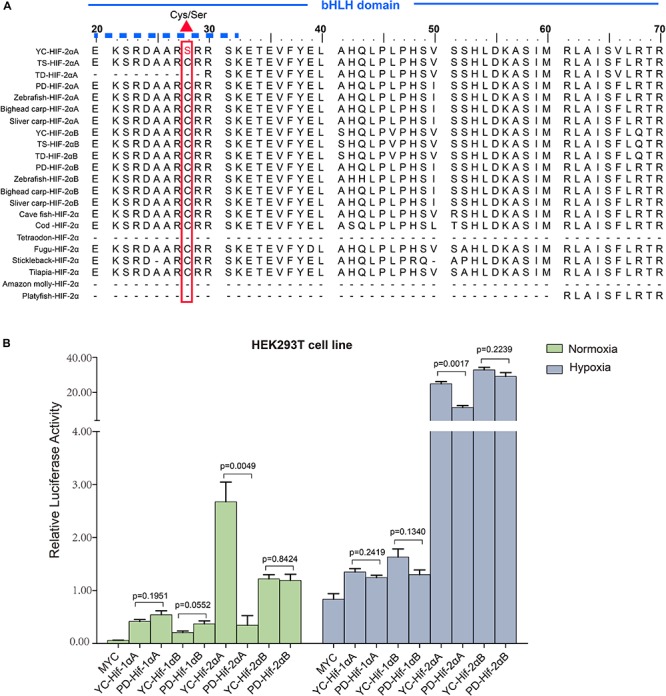
Transcriptional activity analysis of different HIF-αs under normoxic and hypoxic conditions. **(A)** Multiple sequence alignment of the N-terminus of the HIF-2α bHLH domain amino acid sequences. The basic domain that interacts directly with DNA is marked with a solid dash line and the variation between serine and cysteine at position 28 is marked by a regular red triangle. YC, *T. scleroptera*, PD: *P. dabryanus* TD, *T. dalaica*; TS, *T. siluroides*. **(B)** Transcriptional activity comparative analysis of different HIF-αs between high-elevation *T. scleroptera* (YC) and low-elecation *P. dabryanus* (PD). Significant differences in the transcriptional activity of HIF-2αA. Error bars represent the mean ± SEM. Statistical significance was determined using the two-tailed unpaired Student *t*-test with *P* < 0.05.

Our luciferase reporter assay indicated all the HIF-α paralogs could upregulate the luciferase activity of the HRE reporter gene through dimerization with human HIF-β. Different HIF-α forms showed different levels of transcriptional activity. In general, hypoxia could enhance the transcriptional activity of HIF-αs. Intriguingly, in comparison with plain loach, HIF-2αA from the high-land loach exhibited stronger effects on transcription whether under normoxic or hypoxic conditions (*p* < 0.05). However, there were no significant differences among the other HIF-α paralogs (Figure 4B). Subsequently, through multiple comparative analysis of amino acid sequences for HIF-2αA, we found that the only difference in the DNA-binding basic regions was that the cysteine found in all other representative fishes correlated with serine 28 in *T. scleroptera* (Figure 4A). As Okuno mentioned, this nonsynonymous mutation makes cysteines mutate serines, increasing the transcriptional activity by enhance affinity with DNA-binding complex(Okuno et al., 1993).

### YC-VHL Stabilizes the HIF-α Potein and Enhances the Transcriptional Activity of HIF-α Genes

**FIGURE 5:**
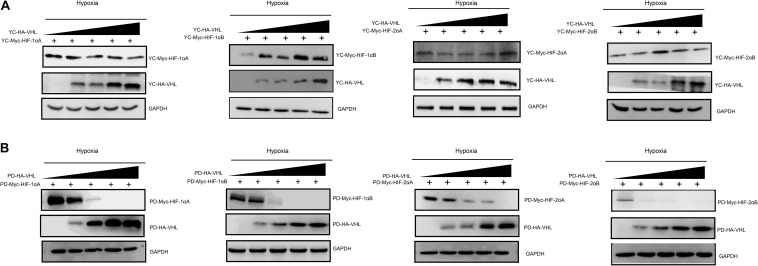
The effects of different concentration gradients pVHL on the protein expression of HIF-αs under hypoxia. **(A)** The effect of YC-VHL on Tibetan loach (YC) HIF-α isoforms (HIF-1αA/B*and* HIF-2αA/B) under hypoxic conditions. **(B)** The effect of PD-VHL on plain loach (PD) HIF-α isoforms under hypoxic conditions.HEK293T cells were co-transfected with equal amounts of Myc-tagged HIF-αs expression vector along with increasing amounts of HA-tagged pVHL expression vector under hypoxia.

**FIGURE 6:**
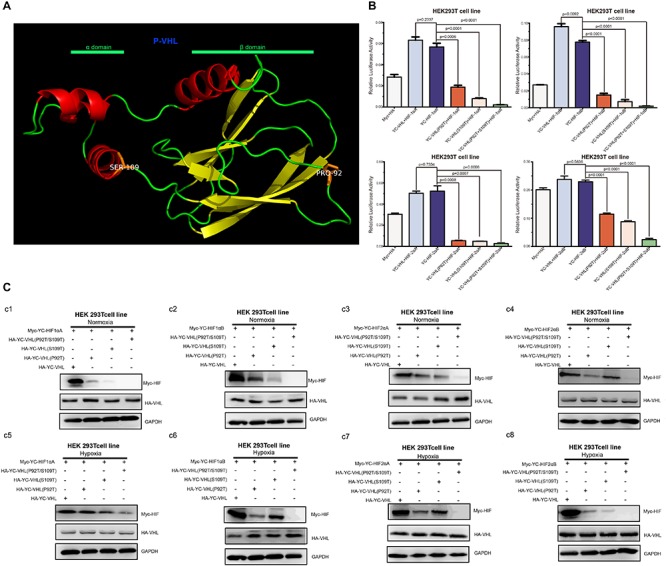
The effects of pVHL mutants’ overexpression in HEK 293T cell on the transcriptional activity and protein expression levels of HIF-αs **(A)** Three-dimensional views of the pVHL protein, highlighting YC-pVHL specific amino acid substitutions. pVHL has two tightly coupled domains consisting of β sandwich (β domain), and three-helix cluster (α domain). Two mutants: Ser109 atoms located in helix cluster, and Pro 92 located in β sandwich. **(B)** The HRE reporter activity induced by hypoxia was suppressed by overexpression of YC-VHL mutant, while overexpression of wide type YC-VHL in HEK293T cells did not affect HRE promoter luciferase reporter activity under hypoxia condition. **(C)** Comparisons of different VHL mutants on the stability of Tibetan loach HIF-αs. (c1–c4) The protein level of HIF-αs by over expression of wide type YC-pVHL and pVHL mutant under normoxia. (c5–c8) The protein level of HIF-αs by over expression of wide type YC pVHL and pVHL mutant under hypoxia. HEK293T cells were transfected with indicated plasmids. After 16–20 h under normoxia (21% O2) or hypoxia (2% O2), cells were harvested and detected by Western blot analysis. Error bars represent the mean ± SEM. Statistical significance was determined using the two-tailed unpaired Student *t*-test with *P* < 0.05.

Hypoxia inducible factor (HIF) -mediated transcriptional activation is the most critical signaling pathway for cells to sense hypoxia, in this process, pVHL is crucial to the stability of HIF-α. In light of the well-understood function of HIF-α in hypoxia signaling and hypoxia adaptation for fishes living at high elevation, we next studied whether *T. scleroptera* pVHL is unique compared to that of species in the plain habitat. Initially, we examined whether pVHL could affect HIF-α transactivity in different ways, by cotransfecting HA-tagged pVHL together with Myc-tagged HIF-α, HRE-luciferase reporter and pRL-Renilla luciferase reporters into HEK 293T cells. Overexpression of PD-VHL significantly inhibited the transactivity of the HIF-α paralogs (Supplementary Figure S5B) under either normoxic or hypoxic condition. In contrast, YC-VHL could considerably suppress the transactivity of HIF-α paralogs under normoxic conditions, whereas YC-VHL did not inhibit the transactivity of HIF-α paralogs, even enhancing the HIF transcriptional activity to some extent under hypoxia (Supplementary Figure S5A). Subsequently, we examined whether YC-VHL affected HIF-α protein expression levels differently than PD-VHL did, the constructed expression plasmid were cotransfected into HEK 293T cells. The results indicated that the expression level of each YC-HIF-α paralog was upregulated by YC-VHL, especially HIF-1αB. In contrast, PD-VHL induced PD-HIF-α degradation (Supplementary Figure S6). To further confirm the effect of each group of pVHL on the respective HIF-α paralogs, we transfected pVHL in a dose-dependent manner, and the results showed that YC-VHL changed HIF-α stability to a negligible degree, and upgraded HIF-α protein expression levels (e.g., HIF-1αB). However, PD-VHL promoted *P.dabryanus* HIF-α degradation in a dose-dependent manner (Figure 5). Through the further inspection of the amino acid sequences for pVHL coupled with selection analysis and 3D structure, we obtained three mutants of YC-VHL that mimic the amino acids of PD-VHL; specifically, Pro at position 92 was mutated to Thr (YC-VHL-P92T), Ser at position 109 was mutated to Thr (YC-VHL-S109T), and the two sites were simultaneously mutated (YC-VHL-P92T + S109T). Luciferase reporter assays revealed that YC-VHL site mutants repressed the HRE reporter activity activated by HIF-α overexpression, while the wide type YC-VHL did not significantly affect HRE-reporter activity under hypoxic conditions (Figure 6B). Furthermore, this result confirmed by western blot assays. YC-VHL mutants could induce HIF-α degradation under hypoxia or normoxia to an extent similar to that of PD-VHL (Figure 6B). These results not only show that the induction of HIF-α degradation owing to the uniqueness of PD-VHL, but also indicate that YC-VHL may uniquely modulate hypoxia signaling, thus promoting hypoxia tolerance by means of stabilizing HIF-α proteins expression and strengthening transcriptional activity.

## Discussion

Animals living on the plateau or in caves, such as birds, mammals and fishes, have been exposed to a low-oxygen environment for a long time (Ramirez et al., 2007). The primary research on hypoxia adaptation mainly focused on endothermic vertebrates. However, studies performed on poikilothermic animals is relatively scarce. The fish fauna of the Qinghai-Tibet Plateau is mainly composed of three groups, namely, the *Schizothoracine* fish, *Glyptosternoids*, and *Triplophysa*, which are considered excellent fish models for studying high-altitude adaptation (Kang et al., 2017). They are all well adapted to the harsh ecological environment of the Qinghai Tibet Plateau, characterized by the cold, hypoxia and strong UV. HIF-α and pVHL, as the key factors in hypoxia signaling pathway, dominating the main signal regulation network governing cells in response to hypoxia (Graham and McCracken, 2019), may diverge between high-altitude and low-elevation species. Although previous studies identified HIF-αs in highland fishes (Cao et al., 2008; Guan et al., 2014), however, all previous studies of fishes focused only on HIF-α sequence analysis or simple functional analysis of individual genes. The present study based on the mechanisms of regulation of HIF-α subunits by pVHL will increase our understanding of the hypoxia signaling pathway along with help us to understand the mechanisms of high-altitude adaptation of the *Triplophysa* fishes to the aquatic fields of the Qinghai-Tibet Plateau.

Environmental heterogeneity affects the adaptive evolutionary trajectory of species. Oxygen capacity often affects the physiological, biochemical and life cycle activities of fishes, and also has a profound impact on both the phenotypes and geographical distributions of living beings. For instance, *Carassius carassius* can survive several months in a cold and hypoxic environment (Nilsson and Ostlund-Nilsson, 2004). In contrast, the Wuchang bream (*Megalobrama amblycephala*) is a hypoxia-sensitive species, hence, even transitory hypoxia treatment at room temperature is fatal (Ouyang et al., 2001). The genus *Triplophysa* endemic to the Tibetan plateau and experience extreme environmental conditions. Therefore, these species present an ideal opportunity to study the hypoxia adaptation of fishes. With comparative analysis of amino acid sequences of multiple species, we found that each HIF-α contained the main domains and the key residues (proline residues) found in their mammalian counterparts (Prabhakar and Semenza, 2012). HIF-1α is structurally similar to HIF-2α; these two subunits share 43% amino acid sequence identity and are mainly differed in the N-TAD domain. HIF-α N-terminus domain (e.g., bHLH-PAS) was extremely conserved, but C-terminus domain (e.g., ODDD) was not conserved, especially the transactivation domains, implying that their products exerted non-redundant function in mediating various responses mechanisms (Kaelin and Ratcliffe, 2008). Phylogenetic analysis showed that HIF-α from a single copy gene to multiple paralogs, as Ohno mentioned, genes or entire genomes duplication can be an efficacious strategy for producing raw genetic material during evolution, which is especially considerable for organisms inhabiting variable surroundings (Ohno, 1971).

Selection analysis showed that among HIF-α genes, only HIF-1αA experienced significantly selective pressure.

We compared HIF-1αA sequences of multiple species to identify site variations and to understand the mechanism of functional evolution of the *Triplophysa* fishes. Five sites (325A, 340N, 591D, 765I, and 770F) that are uniquely present in the high-altitudinal species. Of these variable sites only 340N were detected as positively selected sites (117 L, 131 H, 133 T, 138 S, and 153 L) in the branch-site model, which might be expected to affect the PAS domain folding (Maynard and Ohh, 2004). In a shared highland environment, adaptive selection for chronic hypoxia is a vital unit of the evolution for aboriginal species. Previous studies, including ectothermic snakes (Li et al., 2018), Tibetans (Beall et al., 2010), Tibetan horses (Liu et al., 2019), and Tibetan mastiff (Miao et al., 2016), have identified EPAS1, also named HIF-2α shows the strongest semaphore for positive selection. Given the results of our study together with previous study of schizothoracine fish (Guan et al., 2014), significant selective pressure was detected in HIF-1α. It appears that the fishes have developed distinct mechanisms to adapt the unfavorable ecological environment of the Tibetan Plateau.

Functional analysis was performed by transfection of HIF-αs and pVHL into HEK 293T cells. Different HIF-αs performed different transcriptional activities. Luciferase analyses in the current study revealed that HIF-2αA isoforms more efficiently induced target gene expression. Divergence of transcriptional activity usually been related to differences in N-terminal transactivation domains between HIF-1α and HIF-2α, which imply that intrinsic differences of HIF-α isoforms coding sequences might facilitate isoform-particular function (Koh and Powis, 2012). Generally, during chronic hypoxic exposure, HIF-2α variants plays an important major role in driving the hypoxic reaction (Hu et al., 2003; Koh and Powis, 2012). In comparison with plain loach, Tibetan loach exhibit dramatic increases in the transcription activity of HIF-2αA. The specific nonsynonymous mutation of the conserved cysteines to serines at position 28, yielded a stronger affinity DNA-binding compound, subsequently strengthened cell transforming ability (Lando et al., 2000; Rytkonen et al., 2007). Although the HIF-2α transcriptional activity is higher than HIF-1α, nevertheless the protein expression level appeared antithetic tendency, suggesting HIF-α protein levels are related to posttranscriptional or posttranslational changes (Kietzmann et al., 2016). This result is similar to that found for schizothoracine fish, which also located on the Tibetan Plateau (Guan et al., 2014). By Comparing of protein level degradation rates, we found that HIF-α proteins from *P. dabryanus* (PD) were degraded faster than those of the *T. scleroptera* (YC) in response to hypoxia. *T. scleroptera* HIF-αs are more stable than those of low-elevation loach, leading to accumulation and are thought to enhance cell proliferation, migration, and survival for chronic hypoxia adaptation (Stantic et al., 2018).

HIF-α and pVHL, as the key factors in the hypoxia signaling pathway, might diverge between organisms in high-altitude adaptation and lowland species. After aligning the amino acid sequences of pVHL from different species, we found that some unique amino acid sites were detected as possible positively selected sites (19 A, 61 T, 98 A, 109 S), restricted to the highest-altitudinal species *T. scleroptera.* Notably, proline-to-threonine substitution at position 92, which is located in the β domain might affects protein function as predicted by the PROVEAN. Exploring regulatory mechanisms of HIF-αs by pVHL will improve our understanding of the hypoxia signaling pathway and plateau adaptation. To test the potential functional effects of pVHL in regulating HIF stability, we compared regulatory mechanisms in Tibetan Plateau compared with the lowland species. *T. scleroptera* wild-type pVHL (YC-VHL) did not influence HER-reporter activity under hypoxia. However *P. dabryanus* pVHL (PD-VHL) could decline the transcriptional activity of HIF-αs. Using YC-VHL mutants (P92T, S109T) to mimic amino acids of low elevation allele, our results showed that the pVHL mutant could suppress the transcriptional activity of YC-HIF-αs under hypoxia, suggesting that YC-VHL may uniquely modulate hypoxia signaling. It is worth noting that P/S are all non-polar amino acids whereas T is a polar amino acid and this mutation might leads to protein conformational diversification. The unique feature of YC-VHL may contribute to the specific high-altitude adaptation. Furthermore, we revealed that YC-VHL facilitates hypoxia tolerance by stabilizing HIF-α proteins, whereas PD-VHL could induce HIF-α degradation under hypoxia or normoxia. Notably, Our site-directed mutagenesis experiment further demonstrated that YC-VHL can stabilize HIF-α proteins. These phenomena are consistent with aquatic cetaceans unique feature of pVHL to hypoxia adaptation, which possess divergent diving capabilities with commensurate differences in oxygenation. (Bi et al., 2017). Even though we have boldly explained this phenomenon with some experimental evidences, we cannot preclude the existence of other signal pathways to mediate HIF-α function in hypoxia tolerance (Webb et al., 2009). HIF-α regulation is complicated in fishes and might be shaped by multiple physiological and environmental factors. Further research is indispensable to investigate the structural and functional differences of HIF-α subunits.

## Data Availability Statement

The sequencing data have been deposited into the National Center for Biotechnology Information (GenBank: MN990446–MN990455).

## Ethics Statement

Experiments involving animals in this study were conducted in accordance with the Laboratory Animal Management Principles of China. All experimental protocols were approved by the Ethics Committee of the Institute of Hydrobiology, Chinese Academy of Sciences.

## Author Contributions

SH and LY led the project. JC performed the experiments, analyzed the data, and wrote and revised the manuscript. YS, JW, GO, JK, and WL participated in fish sampling and provided advices in experiments.

## Conflict of Interest

The authors declare that the research was conducted in the absence of any commercial or financial relationships that could be construed as a potential conflict of interest.

## Footnotes

1 http://www.uniprot.org/

2 http://www.ncbi.nlm.nih.gov/genbank/

3 http://www.ensembl.org

## Supplementary Material

FIGURE S1 Multiple sequence alignment of the deduced HIF-1α protein sequences from human, zebrafish, *T. scleroptera* (YC), *P. dabryanus* (PD). Dashes indicate the gaps inserted to facilitate alignment. The main domain of HIF-1αA/B is marked with a solid line box or an overline. The two conserved proline residues within the ODDD domain and the asparagine residue in C-TAD are indicated by the red arrows. The gray boxes represent two conserved proline hydroxylation motif LxxLAP areas. The DNA-interacting basic amino acids K19, K21, R27, R29, R30, D55, and K56 are highlighted in green.

FIGURE S2 Multiple sequence alignment of the deduced HIF-2α protein sequences from human, zebrafish, *T. scleroptera* (YC), *P. dabryanus* (PD). Dashes indicate the gaps inserted to facilitate alignment. The main domain of HIF-1αA/B is marked with a solid line box or an overline. The two conserved proline residues within the ODDD domain and the asparagine residue in C-TAD are indicated by the red arrows. The gray boxes represent two conserved proline hydroxylation motif LxxLAP areas. The DNA-interacting basic amino acids K16, K18, R24, R26, R27, D52, and K53 are highlighted in green.

FIGURE S3 Domain structure of hypoxia-inducible factor subunits. **(A)** Domain structure of HIF-1αB. **(B)** Domain structure of HIF-2αA. **(C)** Domain structure of HIF-2αB. The following domains are shown: basic helix-loop-helix domain (bHLH), Per-Arnt-Sim homology domain (PAS-A/B), a PAS-associated COOH-terminal (PAC) motif, O2-dependent degradation domain (ODDD), NH2- and COOH-terminal transactivation domains (TAD-N and TAD-C). Percentage represents the similarity ratio of the domain between highland loach (YC: *T. scleroptera*) and plain loach (PD: *P. dabryanus*). Amino acid replacements in *Triplophysa* compared with all low-elevation species are shown in red. TD, *T. dalaica*; TS, *T. siluroides*.

FIGURE S4 Sequence and domain analysis of pVHL **(A)** Multiple sequence alignment of the deduced pVHL protein sequences. The red underline indicates β domain, the blue dotted line represents the α domain. Amino acid replacements in *T.*compared with *P. dabryanus* (PD) marked with red box. The red star marks the amino acid replacement that affects protein function as predicted by PROVEAN. **(B)** A maximum-likelihood tree and alignment of fish pVHL sequences. Amino acids unique to the YC are highlighted in red, which identified by branch-site model with a posterior probability greater than 0.5.

FIGURE S5 The effects of pVHL overexpression in HEK 293T cell on the transcriptional activity of **(A)** HIF-αs in *T. scleroptera* (YC), and (B) *P. dabryanus* (PD) measured with luciferase activity assays. ^∗^*p* < 0.05, ^∗∗^*p* < 0.01, ^∗∗∗^*p* < 0.001, ns represents no significant difference. Error bars indicate the standard error of the mean.

FIGURE S6 The effects of pVHL overexpression in HEK 293T cell on the protein expression of (a1,a2) HIF-αs in *T. scleroptera* (YC) under normal normoxia and hypoxia conditions (a3,a4) HIF-αs in *P. dabryanus* (PD) under normal normoxia and hypoxia conditions. HEK293T cells were co-transfected with equal amounts of Myc-tagged HIF-αs expression vector along with HA-tagged pVHL expression vector.

TABLE S1 The PCR primers used to amplify *T. scleroptera* and *P. dabryanus.*

TABLE S2 Gene specific primers with restriction enzyme sites for full-length pVHL and HIF-α isoforms.

TABLE S3 Gene information of *T. scleroptera* and *P. dabryanus.*

TABLE S4 Comparison amino acid sequence similarity of pVHL and HIF-α isoforms between *T. scleroptera* and other species.

TABLE S5 Amino acid variations of pVHL gene *in T. scleroptera.*

TABLE S6 List of HIF-α genes in representive invertebrates and vertebrates. The black filled pentagram and empty pentagram represent the existency and inexistence of HIF gene in the selected species.

TABLE S7 One-ratio model analysis of HIF-αs and pVHL gene.

TABLE S8 Selective pressure analyses of hypoxia-inducible factor and pVHL using PAML Branch-Site Model.

## References

[B1] Beall C. M. Cavalleri G. L. Deng L. Elston R. C. Gao Y. Knight J. (2010). Natural selection on EPAS1 (HIF2alpha) associated with low hemoglobin concentration in Tibetan highlanders. *Proc. Natl. Acad. Sci. U.S.A.* 107 11459–11464. 10.1073/pnas.1002443107 20534544 PMC2895075

[B2] Bi J. Hu B. Wang J. Liu X. Zheng J. Wang D. (2017). Beluga whale pVHL enhances HIF-2alpha activity via inducing HIF-2alpha proteasomal degradation under hypoxia. *Oncotarget* 8 42272–42287. 10.18632/oncotarget.15038 28178687 PMC5522066

[B3] Bickler P. E. Buck L. T. (2007). Hypoxia tolerance in reptiles, amphibians, and fishes: life with variable oxygen availability. *Annu. Rev. Physiol.* 69 145–170. 10.1146/annurev.physiol.69.031905.162529 17037980

[B4] Bigham A. W. Lee F. S. (2014). Human high-altitude adaptation: forward genetics meets the HIF pathway. *Genes Dev.* 28 2189–2204. 10.1101/gad.250167.114 25319824 PMC4201282

[B5] Bordoli L. Kiefer F. Arnold K. Benkert P. Battey J. Schwede T. (2009). Protein structure homology modeling using SWISS-MODEL workspace. *Nat. Protoc.* 4 1–13. 10.1038/nprot.2008.197 19131951

[B6] Burga A. Wang W. Ben-David E. Wolf P. C. Ramey A. M. Verdugo C. (2017). A genetic signature of the evolution of loss of flight in the Galapagos cormorant. *Science* 356:eaal3345. 10.1126/science.aal3345 28572335 PMC5567675

[B7] Cao Y. B. Chen X. Q. Wang S. Wang Y. X. Du J. Z. (2008). Evolution and regulation of the downstream gene of hypoxia-inducible factor-1alpha in naked carp (Gymnocypris przewalskii) from Lake Qinghai, China. *J. Mol. Evol.* 67 570–580. 10.1007/s00239-008-9175-4 18941827

[B8] Cheviron Z. A. Brumfield R. T. (2012). Genomic insights into adaptation to high-altitude environments. *Heredity* 108 354–361. 10.1038/hdy.2011.85 21934702 PMC3313048

[B9] Choi Y. Sims G. E. Murphy S. Miller J. R. Chan A. P. (2012). Predicting the functional effect of amino acid substitutions and indels. *PLoS ONE* 7:e46688. 10.1371/journal.pone.0046688 23056405 PMC3466303

[B10] Cockman M. E. Masson N. Mole D. R. Jaakkola P. Chang G. W. Clifford S. C. (2000). Hypoxia inducible factor-alpha binding and ubiquitylation by the von Hippel-Lindau tumor suppressor protein. *J. Biol. Chem.* 275 25733–25741. 10.1074/jbc.M002740200 10823831

[B11] Edgar R. C. (2004). MUSCLE: multiple sequence alignment with high accuracy and high throughput. *Nucleic Acids Res.* 32 1792–1797. 10.1093/nar/gkh340 15034147 PMC390337

[B12] Epstein A. C. R. Gleadle J. M. McNeill L. A. Hewitson K. S. O’Rourke J. Mole D. R. (2001). C-elegans EGL-9 and mammalian homologs define a family of dioxygenases that regulate HIF by prolyl hydroxylation. *Cell* 107 43–54. 10.1016/S0092-8674(01)00507-4 11595184

[B13] Gou X. Wang Z. Li N. Qiu F. Xu Z. Yan D. (2014). Whole-genome sequencing of six dog breeds from continuous altitudes reveals adaptation to high-altitude hypoxia. *Genome Res.* 24 1308–1315. 10.1101/gr.171876.113 24721644 PMC4120084

[B14] Graham A. M. McCracken K. G. (2019). Convergent evolution on the hypoxia-inducible factor (HIF) pathway genes EGLN1 and EPAS1 in high-altitude ducks. *Heredity* 122 819–832. 10.1038/s41437-018-0173-z 30631144 PMC6781116

[B15] Guan L. Chi W. Xiao W. Chen L. He S. (2014). Analysis of hypoxia-inducible factor alpha polyploidization reveals adaptation to Tibetan Plateau in the evolution of schizothoracine fish. *BMC Evol. Biol.* 14:192. 10.1186/s12862-014-0192-1 25205386 PMC4162920

[B16] Herzenstein S. M. (1888). “Fische,” in Wissenschaftliche Resultate der von N. M. Przewalski nach Central-Asien unternommenen Reisen. Zoologischer Theil (St. Petersburg: Kaiserlichen Akademie der Wissenschaften), 54–57.

[B17] Hu C. J. Wang L. Y. Chodosh L. A. Keith B. Simon M. C. (2003). Differential roles of hypoxia-inducible factor 1alpha (HIF-1alpha) and HIF-2alpha in hypoxic gene regulation. *Mol. Cell. Biol.* 23 9361–9374. 14645546 10.1128/MCB.23.24.9361-9374.2003 PMC309606

[B18] Huelsenbeck J. P. Ronquist F. (2001). MRBAYES: bayesian inference of phylogenetic trees. *Bioinformatics* 17 754–755. 10.1093/bioinformatics/17.8.754 11524383

[B19] Ivan M. Kondo K. Yang H. Kim W. Valiando J. Ohh M. (2001). HIFalpha targeted for VHL-mediated destruction by proline hydroxylation: implications for O2 sensing. *Science* 292 464–468. 10.1126/science.1059817 11292862

[B20] Jaakkola P. Mole D. R. Tian Y. M. Wilson M. I. Gielbert J. Gaskell S. J. (2001). Targeting of HIF-alpha to the von Hippel-Lindau ubiquitylation complex by O2-regulated prolyl hydroxylation. *Science* 292 468–472. 10.1126/science.1059796 11292861

[B21] Kaelin W. G. Jr. Ratcliffe P. J. (2008). Oxygen sensing by metazoans: the central role of the HIF hydroxylase pathway. *Mol. Cell.* 30 393–402. 10.1016/j.molcel.2008.04.009 18498744

[B22] Kang J. Ma X. He S. (2017). Evidence of high-altitude adaptation in the glyptosternoid fish, *Creteuchiloglanis macropterus* from the Nujiang River obtained through transcriptome analysis. *BMC Evol Biol.* 17:229. 10.1186/s12862-017-1074-0 29169322 PMC5701497

[B23] Kietzmann T. Mennerich D. Dimova E. Y. (2016). Hypoxia-inducible factors (HIFs) and phosphorylation: impact on stability, localization, and transactivity. *Front. Cell Dev. Biol.* 4:11 10.3389/fcell.2016.00011 PMC4763087 26942179

[B24] Koh M. Y. Powis G. (2012). Passing the baton: the HIF switch. *Trends Biochem. Sci.* 37 364–372. 10.1016/j.tibs.2012.06.004 22818162 PMC3433036

[B25] Lando D. Pongratz I. Poellinger L. Whitelaw M. L. (2000). A redox mechanism controls differential DNA binding activities of hypoxia-inducible factor (HIF) 1alpha and the HIF-like factor. *J. Biol. Chem.* 275 4618–4627. 10.1074/jbc.275.7.4618 10671489

[B26] Letunic I. Doerks T. Bork P. (2015). SMART: recent updates, new developments and status in 2015. *Nucleic Acids Res.* 43 D257–D260. 10.1093/nar/gku949 25300481 PMC4384020

[B27] Li J.-T. Gao Y.-D. Xie L. Deng C. Shi P. Guan M.-L. (2018). Comparative genomic investigation of high-elevation adaptation in ectothermic snakes. *Proc. Natl. Acad. Sci. U.S.A.* 115 8406–8411. 10.1073/pnas.1805348115 30065117 PMC6099860

[B28] Liu X. Zhang Y. Li Y. Pan J. Wang D. Chen W. (2019). EPAS1 gain-of-function mutation contributes to high-altitude adaptation in Tibetan horses. *Mol. Biol. Evol.* 10.1093/molbev/msz158 [Epub ahead of print]. 31273382 PMC6805228

[B29] Ma X. Dai W. Kang J. Yang L. He S. (2015). Comprehensive transcriptome analysis of six catfish species from an altitude gradient reveals adaptive evolution in tibetan fishes. *G3 (Bethesda)* 6 141–148. 10.1534/g3.115.024448 26564948 PMC4704712

[B30] Maynard M. A. Ohh M. (2004). von Hippel-Lindau tumor suppressor protein and hypoxia-inducible factor in kidney cancer. *Am. J. Nephrol.* 24 1–13. 10.1159/000075346 14654728

[B31] Meyer A. Van de Peer Y. (2005). From 2R to 3R: evidence for a fish-specific genome duplication (FSGD). *Bioessays* 27 937–945. 10.1002/bies.20293 16108068

[B32] Miao B. Wang Z. Li Y. (2016). Genomic analysis reveals hypoxia adaptation in the Tibetan mastiff by introgression of the gray wolf from the Tibetan Plateau. *Mol. Biol. Evol.* 34 734–743. 10.1093/molbev/msw274 27927792

[B33] Nilsson G. E. Ostlund-Nilsson S. (2004). Hypoxia in paradise: widespread hypoxia tolerance in coral reef fishes. *Proc. Biol. Sci.* 271 (Suppl. 3), S30–S33. 10.1098/rsbl.2003.0087 15101411 PMC1810002

[B34] Ohno S. (1971). *Evolution by Gene Duplication.* Berlin: Springer Science & Business Media.

[B35] Okuno H. Akahori A. Sato H. Xanthoudakis S. Curran T. Iba H. (1993). Escape from redox regulation enhances the transforming activity of fos. *Oncogene* 8 695–701. 8437852

[B36] Opazo J. C. Butts G. T. Nery M. F. Storz J. F. Hoffmann F. G. (2013). Whole-genome duplication and the functional diversification of teleost fish hemoglobins. *Mol. Biol. Evol.* 30 140–153. 10.1093/molbev/mss212 22949522 PMC3525417

[B37] Ouyang M. Yu X. Chen D. Y. (2001). The preliminary studies on oxygen consumption rate and asphyxia point ofMegalobrama amblycephalain Poyang Lake. *J. Jiangxi Fish. Sci. Technol.* 4 20–22.

[B38] Posada D. (2008). jModelTest: phylogenetic model averaging. *Mol. Biol. Evol.* 25 1253–1256. 10.1093/molbev/msn083 18397919

[B39] Prabhakar N. R. Semenza G. L. (2012). Adaptive and maladaptive cardiorespiratory responses to continuous and intermittent hypoxia mediated by hypoxia-inducible factors 1 and 2. *Physiol. Rev.* 92 967–1003. 10.1152/physrev.00030.2011 22811423 PMC3893888

[B40] Qi D. Chao Y. Guo S. Zhao L. Li T. Wei F. (2012). Convergent, parallel and correlated evolution of trophic morphologies in the subfamily schizothoracinae from the Qinghai-Tibetan plateau. *PLoS ONE* 7:e34070. 10.1371/journal.pone.0034070 22470515 PMC3314705

[B41] Rahman M. S. Thomas P. (2007). Molecular cloning, characterization and expression of two hypoxia-inducible factor alpha subunits, HIF-1alpha and HIF-2alpha, in a hypoxia-tolerant marine teleost, Atlantic croaker (Micropogonias undulatus). *Gene* 396 273–282. 10.1016/j.gene.2007.03.009 17467194

[B42] Ramirez J. M. Folkow L. P. Blix A. S. (2007). Hypoxia tolerance in mammals and birds: from the wilderness to the clinic. *Annu. Rev. Physiol.* 69 113–143. 10.1146/annurev.physiol.69.031905.163111 17037981

[B43] Raval R. R. Lau K. W. Tran M. G. Sowter H. M. Mandriota S. J. Li J. L. (2005). Contrasting properties of hypoxia-inducible factor 1 (HIF-1) and HIF-2 in von Hippel-lindau-associated renal cell carcinoma. *Mol. Cell. Biol.* 25 5675–5686. 10.1128/MCB.25.13.5675-5686.2005 15964822 PMC1157001

[B44] Rytkonen K. T. Akbarzadeh A. Miandare H. K. Kamei H. Duan C. Leder E. H. (2013). Subfunctionalization of cyprinid hypoxia-inducible factors for roles in development and oxygen sensing. *Evolution* 67 873–882. 10.1111/j.1558-5646.2012.01820.x 23461336

[B45] Rytkonen K. T. Vuori K. A. Primmer C. R. Nikinmaa M. (2007). Comparison of hypoxia-inducible factor-1 alpha in hypoxia-sensitive and hypoxia-tolerant fish species. *Compar. Biochem. Physiol. Part D Genomics Proteomics* 2 177–186. 10.1016/j.cbd.2007.03.001 20483291

[B46] Scheinfeldt L. B. Tishkoff S. A. (2010). Living the high life: high-altitude adaptation. *Genome Biol.* 11:133. 10.1186/gb-2010-11-9-133 20979669 PMC2965377

[B47] Schofield C. J. Ratcliffe P. J. (2004). Oxygen sensing by HIF hydroxylases. *Nat. Rev. Mol. Cell Biol.* 5 343–354. 10.1038/nrm1366 15122348

[B48] Schrodinger L. L. C. (2010). *The PyMOL Molecular Graphics System, Version 1.3r1.* Available online at: http://www.pymol.org

[B49] Semenza G. L. (1999). Regulation of mammalian O2 homeostasis by hypoxia-inducible factor 1. *Annu. Rev. Cell Dev. Biol.* 15 551–578. 10.1146/annurev.cellbio.15.1.551 10611972

[B50] Semenza G. L. (2012). Hypoxia-inducible factors in physiology and medicine. *Cell* 148 399–408. 10.1016/j.cell.2012.01.021 22304911 PMC3437543

[B51] Shams I. Avivi A. Nevo E. (2004). Hypoxic stress tolerance of the blind subterranean mole rat: expression of erythropoietin and hypoxia-inducible factor 1 alpha. *Proc. Natl. Acad. Sci. U.S.A.* 101 9698–9703. 10.1073/pnas.0403540101 15210955 PMC470738

[B52] Soitamo A. J. Rabergh C. M. Gassmann M. Sistonen L. Nikinmaa M. (2001). Characterization of a hypoxia-inducible factor (HIF-1alpha) from rainbow trout-accumulation of protein occurs at normal venous oxygen tension. *J. Biol. Chem.* 276 19699–19705. 10.1074/jbc.M009057200 11278461

[B53] Stantic M. Wolfsberger J. Sakil H. A. M. Wilhelm M. T. (2018). DeltaNp73 enhances HIF-1alpha protein stability through repression of the ECV complex. *Oncogene* 37 3729–3739. 10.1038/s41388-018-0195-2 29628507 PMC6033838

[B54] Stebbins C. E. Kaelin W. G. Jr. Pavletich N. P. (1999). Structure of the VHL-ElonginC-ElonginB complex: implications for VHL tumor suppressor function. *Science* 284 455–461. 10.1126/science.284.5413.455 10205047

[B55] Su J. Wang Z. (1992). Studies on the population energetics of plateau zokor: average daily metabolic rate and burrowing metabolic rate. *Acta TheriolSin* 12 200–206.

[B56] Tarade D. Lee J. E. Ohh M. (2019). Evolution of metazoan oxygen-sensing involved a conserved divergence of VHL affinity for HIF1α and HIF2α. *Nat. Commun.* 10 3293. 10.1038/s41467-019-11149-1 31337753 PMC6650433

[B57] Terova G. Rimoldi S. Corà S. Bernardini G. Gornati R. Saroglia M. (2008). Acute and chronic hypoxia affects HIF-1α mRNA levels in sea bass (Dicentrarchus labrax). *Aquaculture* 279 150–159. 10.1016/j.aquaculture.2008.03.041

[B58] Tian R. Wang Z. Niu X. Zhou K. Xu S. Yang G. (2016). Evolutionary genetics of hypoxia tolerance in cetaceans during diving. *Genome Biol. Evol.* 8 827–839. 10.1093/gbe/evw037 26912402 PMC4824146

[B59] Wang G. L. Jiang B. H. Rue E. A. Semenza G. L. (1995). Hypoxia-inducible factor 1 is a basic-helix-loop-helix-PAS heterodimer regulated by cellular O2 tension. *Proc. Natl. Acad. Sci. U.S.A.* 92 5510–5514. 10.1073/pnas.92.12.5510 7539918 PMC41725

[B60] Wang G. L. Semenza G. L. (1993). General Involvement of Hypoxia-Inducible Factor-I in transcriptional response to hypoxia. *Proc. Natl. Acad. Sci. U.S.A.* 90 4304–4308. 10.1073/pnas.90.9.4304 8387214 PMC46495

[B61] Wang Y. Shen Y. Feng C. Zhao K. Song Z. Zhang Y. (2016). Mitogenomic perspectives on the origin of Tibetan loaches and their adaptation to high altitude. *Sci Rep* 6:29690. 10.1038/srep29690 27417983 PMC4945904

[B62] Wang Y. Yang L. Wu B. Song Z. He S. (2015a). Transcriptome analysis of the plateau fish (*Triplophysa dalaica*): implications for adaptation to hypoxia in fishes. *Gene* 565 211–220. 10.1016/j.gene.2015.04.023 25869933

[B63] Wang Y. Yang L. Zhou K. Zhang Y. Song Z. He S. (2015b). Evidence for adaptation to the tibetan plateau inferred from tibetan loach transcriptomes. *Genome Biol. Evol.* 7 2970–2982. 10.1093/gbe/evv192 26454018 PMC5635588

[B64] Wang Z. Yonezawa T. Liu B. Ma T. Shen X. Su J. (2011). Domestication relaxed selective constraints on the yak mitochondrial genome. *Mol. Biol. Evol.* 28 1553–1556. 10.1093/molbev/msq336 21156878

[B65] Webb J. D. Coleman M. L. Pugh C. W. (2009). Hypoxia, hypoxia-inducible factors (HIF), HIF hydroxylases and oxygen sensing. *Cell Mol. Life. Sci* 66 3539–3554. 10.1007/s00018-009-0147-7 19756382 PMC11115642

[B66] Wu Y. Wu C. (1992). *The Fishes of the Qinghai-Xizang Plateau.* Chengdu: Sichuan Science and Technology Press.

[B67] Xu Q. Zhang C. Zhang D. Jiang H. Peng S. Liu Y. (2016). Analysis of the erythropoietin of a Tibetan Plateau schizothoracine fish (*Gymnocypris dobula*) reveals enhanced cytoprotection function in hypoxic environments. *BMC Evol. Biol.* 16:11. 10.1186/s12862-015-0581-0 26768152 PMC4714423

[B68] Xu S. Li S. Yang Y. Tan J. Lou H. Jin W. (2011). A genome-wide search for signals of high-altitude adaptation in Tibetans. *Mol. Biol. Evol.* 28 1003–1011. 10.1093/molbev/msq277 20961960

[B69] Yang L. Wang Y. Zhang Z. He S. (2014). Comprehensive transcriptome analysis reveals accelerated genic evolution in a Tibet fish, *Gymnodiptychus pachycheilus*. *Genome Biol. Evol.* 7 251–261. 10.1093/gbe/evu279 25543049 PMC4316632

[B70] Yang Z. Wong W. S. Nielsen R. (2005). Bayes empirical bayes inference of amino acid sites under positive selection. *Mol. Biol. Evol.* 22 1107–1118. 10.1093/molbev/msi097 15689528

[B71] Yang Z. H. (2007). PAML 4: phylogenetic analysis by maximum likelihood. *Mol. Biol. Evol.* 24 1586–1591. 10.1093/molbev/msm088 17483113

[B72] Zhang D. Yu M. Hu P. Peng S. Liu Y. Li W. (2017). Genetic adaptation of schizothoracine fish to the phased uplifting of the qinghai-tibetan plateau. *G3 (Bethesda)* 7 1267–1276. 10.1534/g3.116.038406 28209761 PMC5386875

[B73] Zhang W. Fan Z. Han E. Hou R. Zhang L. Galaverni M. (2014). Hypoxia adaptations in the grey wolf (*Canis lupus* chanco) from Qinghai-Tibet Plateau. *PLoS Genet.* 10:e1004466. 10.1371/journal.pgen.1004466 25078401 PMC4117439

[B74] Zhou S. Z. Wang X. L. Wang J. Xu L. B. (2006). A preliminary study on timing of the oldest *Pleistocene glaciation* in Qinghai-Tibetan Plateau. *Quat. Int.* 154 44–51. 10.1016/j.quaint.2006.02.002

