# Supplementary figures and images for: Analysis of Multiplicity of Hypoxia-Inducible Factors in the Evolution of Triplophysa Fish (Osteichthyes: Nemacheilinae) Reveals Hypoxic Environments Adaptation to Tibetan Plateau
Source: Front Genet. 2020 May 12;11:433. doi: 10.3389/fgene.2020.00433 (PMC7235411; doi:10.3389/fgene.2020.00433)

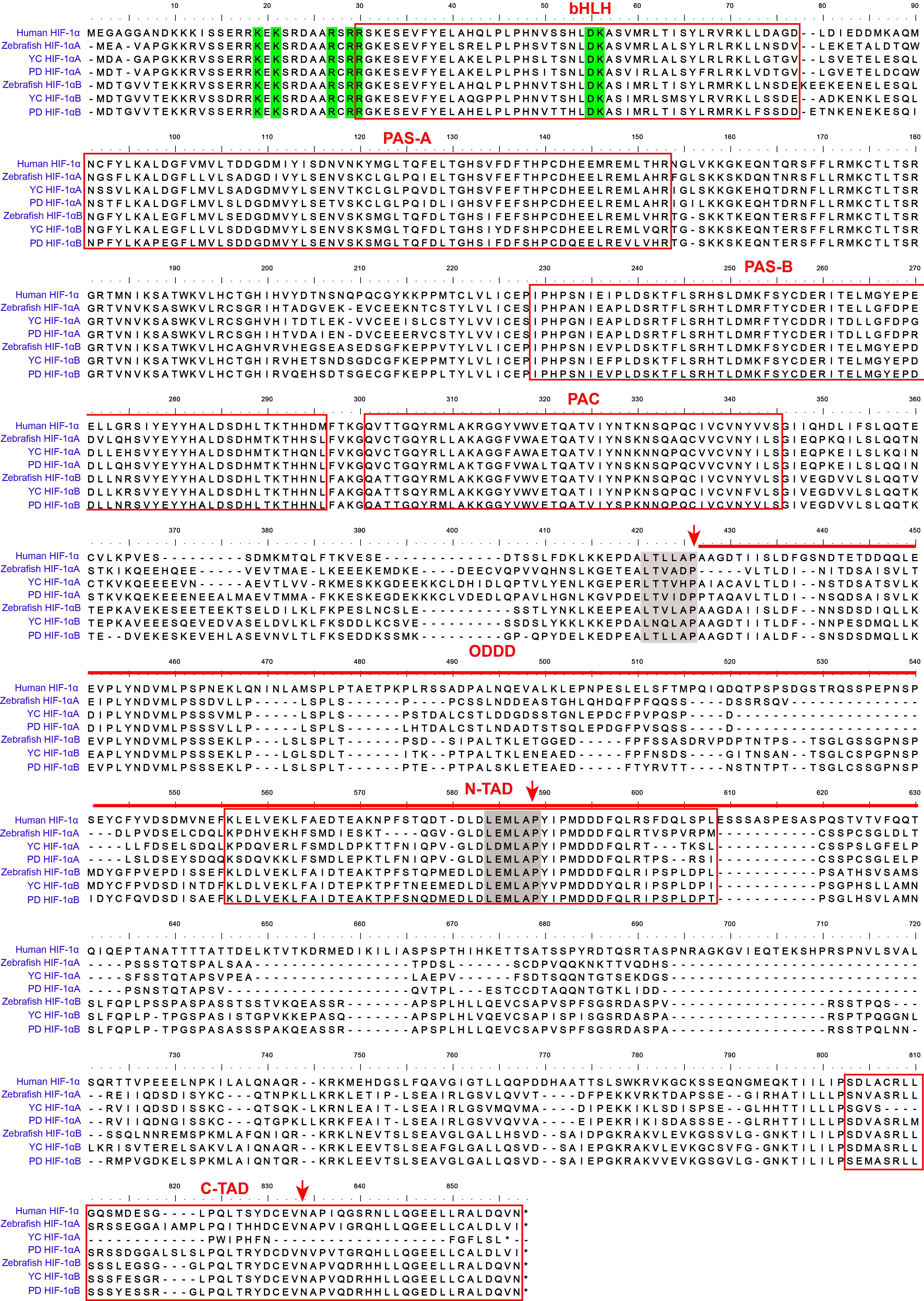

Supplement: FIGURE S1 — Multiple sequence alignment of the deduced HIF-1α protein sequences from human, zebrafish, T. scleroptera (YC), P. dabryanus (PD). Dashes indicate the gaps inserted to facilitate alignment. The main domain of HIF-1αA/B is marked with a solid line box or an overline. The two conserved proline residues within the ODDD domain and the asparagine residue in C-TAD are indicated by the red arrows. The gray boxes represent two conserved proline hydroxylation motif LxxLAP areas. The DNA-interacting basic amino acids K19, K21, R27, R29, R30, D55, and K56 are highlighted in green. [file Image_1.JPEG]

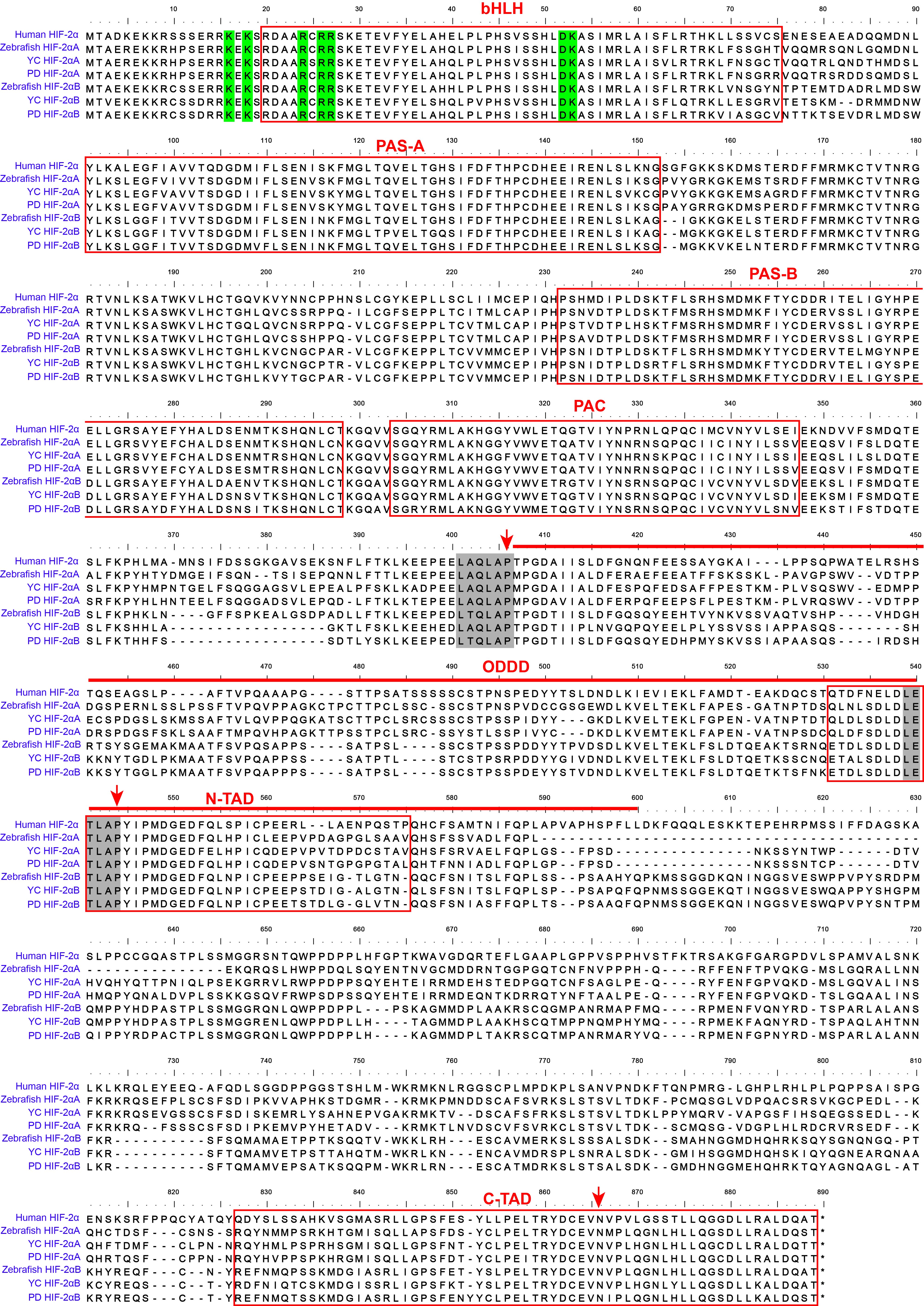

Supplement: FIGURE S2 — Multiple sequence alignment of the deduced HIF-2α protein sequences from human, zebrafish, T. scleroptera (YC), P. dabryanus (PD). Dashes indicate the gaps inserted to facilitate alignment. The main domain of HIF-1αA/B is marked with a solid line box or an overline. The two conserved proline residues within the ODDD domain and the asparagine residue in C-TAD are indicated by the red arrows. The gray boxes represent two conserved proline hydroxylation motif LxxLAP areas. The DNA-interacting basic amino acids K16, K18, R24, R26, R27, D52, and K53 are highlighted in green. [file Image_2.JPEG]

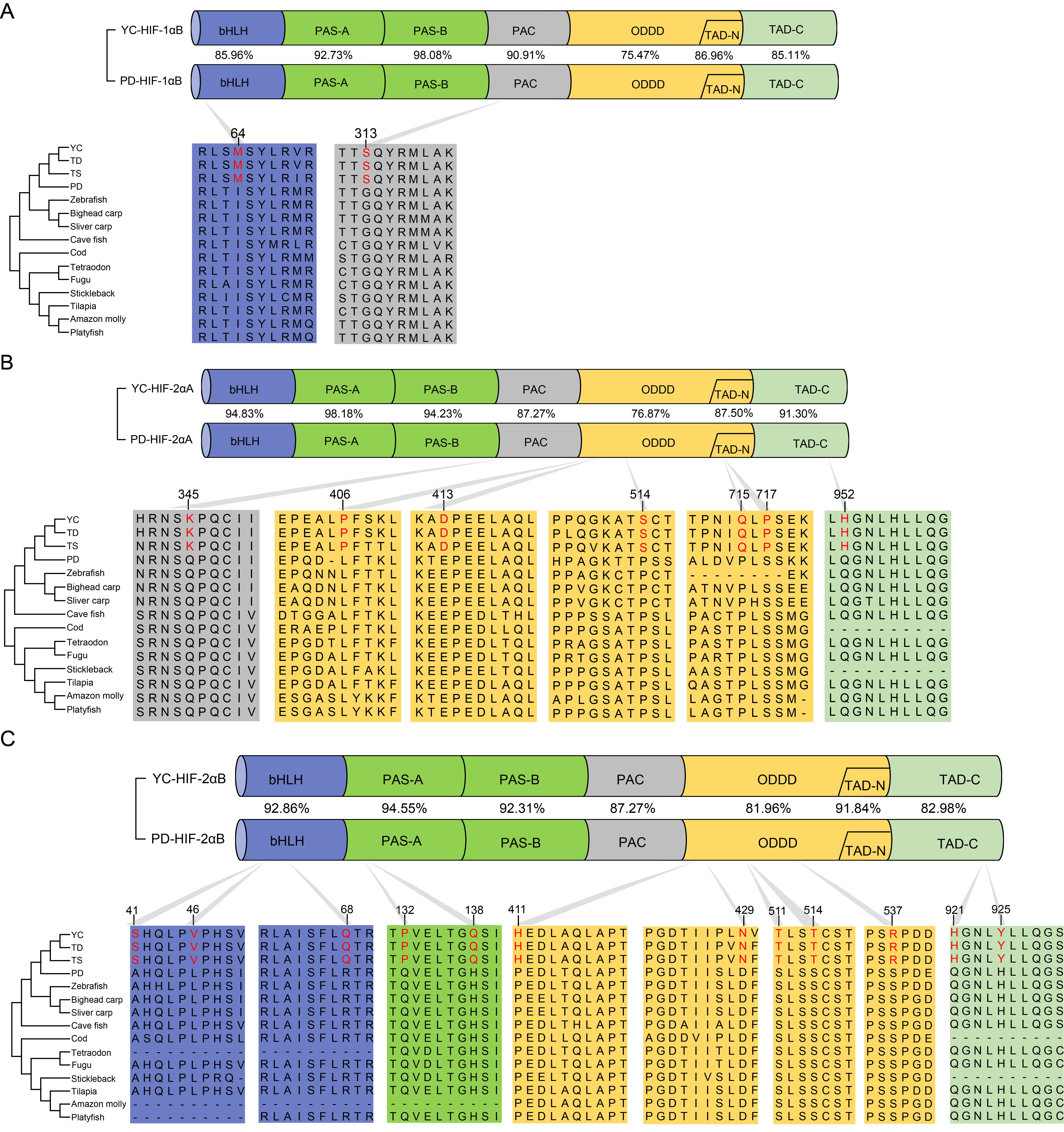

Supplement: FIGURE S3 — Domain structure of hypoxia-inducible factor subunits. (A) Domain structure of HIF-1αB. (B) Domain structure of HIF-2αA. (C) Domain structure of HIF-2αB. The following domains are shown: basic helix-loop-helix domain (bHLH), Per-Arnt-Sim homology domain (PAS-A/B), a PAS-associated COOH-terminal (PAC) motif, O2-dependent degradation domain (ODDD), NH2- and COOH-terminal transactivation domains (TAD-N and TAD-C). Percentage represents the similarity ratio of the domain between highland loach (YC: T. scleroptera) and plain loach (PD: P. dabryanus). Amino acid replacements in Triplophysa compared with all low-elevation species are shown in red. TD, T. dalaica; TS, T. siluroides. [file Image_3.JPEG]

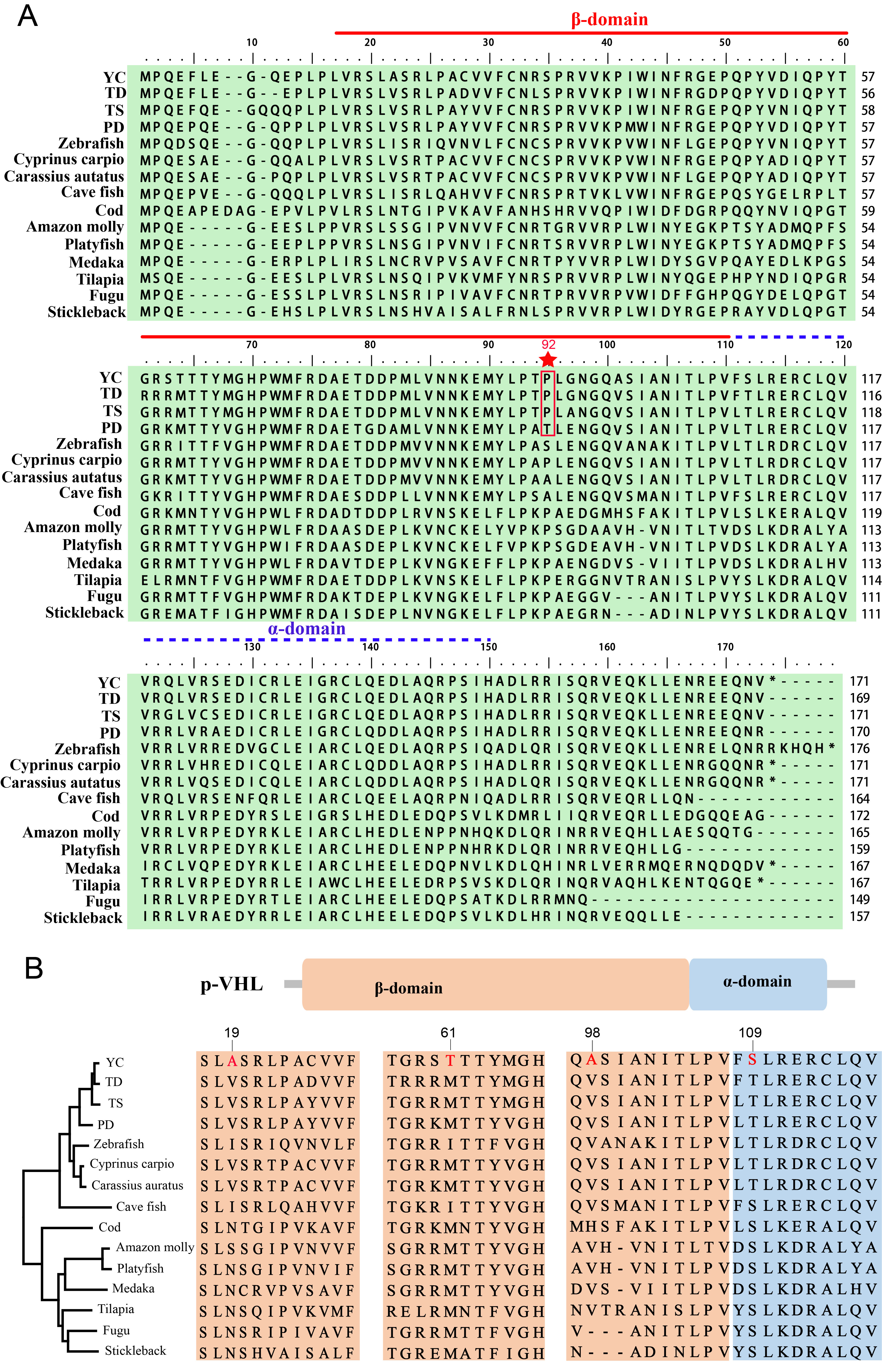

Supplement: FIGURE S4 — Sequence and domain analysis of pVHL (A) Multiple sequence alignment of the deduced pVHL protein sequences. The red underline indicates β domain, the blue dotted line represents the α domain. Amino acid replacements in T.compared with P. dabryanus (PD) marked with red box. The red star marks the amino acid replacement that affects protein function as predicted by PROVEAN. (B) A maximum-likelihood tree and alignment of fish pVHL sequences. Amino acids unique to the YC are highlighted in red, which identified by branch-site model with a posterior probability greater than 0.5. [file Image_4.JPEG]

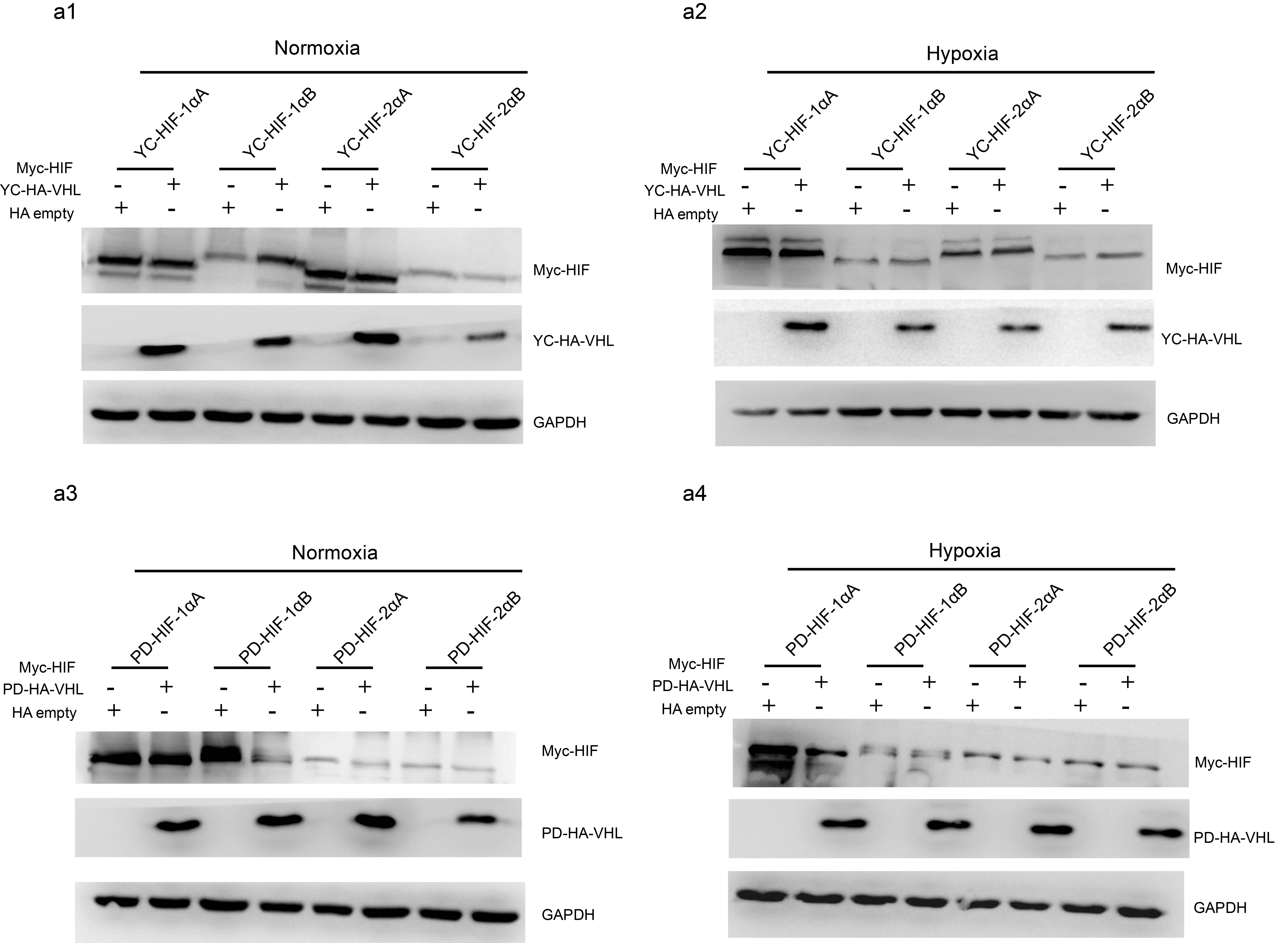

Supplement: FIGURE S6 — The effects of pVHL overexpression in HEK 293T cell on the protein expression of (a1,a2) HIF-αs in T. scleroptera (YC) under normal normoxia and hypoxia conditions (a3,a4) HIF-αs in P. dabryanus (PD) under normal normoxia and hypoxia conditions. HEK293T cells were co-transfected with equal amounts of Myc-tagged HIF-αs expression vector along with HA-tagged pVHL expression vector. [file Image_6.JPEG]
