# Supplementary material for: Analysis of Multiplicity of Hypoxia-Inducible Factors in the Evolution of Triplophysa Fish (Osteichthyes: Nemacheilinae) Reveals Hypoxic Environments Adaptation to Tibetan Plateau
Source: Front Genet. 2020 May 12;11:433. doi: 10.3389/fgene.2020.00433 (PMC7235411; doi:10.3389/fgene.2020.00433)
Supplement: TABLE S1 — The PCR primers used to amplify T. scleroptera and P. dabryanus. [file Table_1.DOCX]

**Table S1 The PCR primers used to amplify *Triplophysa scleroptera* and *Paramisgurnus dabryanus.***

| **Species** | **Gene** | **Primer name** | **Sequence** |
| --- | --- | --- | --- |
| *Triplophysa scleroptera* | VHL | YC-F1 | ACTGGTTGTCTAGGCCATTTCA |
|  |  | YC-R1 | GGTTTCAGGTTTGTCACATTTTACT |
|  | HIF1α-A | YC-F2 | CCATTGCTGATTTAGGATTG |
|  |  | YC-R2 | TGACAACATTCACAAGGATAC |
|  | HIF1α-B | YC-F3 | ACTACAACCGAGTGATGCC |
|  |  | YC-R3 | GCAGGACACTGACTTACTATCT |
|  | HIF2α-A | YC-F4 | GAAAATGGGCATTACGGAG |
|  |  | YC-R4 | GGAACAATCAGAGAGTGGATG |
|  | HIF2α-B | F5-UPM | GAAGATCTTCAGGACTGTAGCCTATCAACTC |
|  |  | F5-USP | GTAAACTTCATGTCCATGCTGTGGC |
|  |  | R5-UPM | ACAGGGCATCTGAAGGTGTGTAACG |
|  |  | R5-USP | AGCCCATCCCACATCCCTCA |
| *Paramisgurnus dabryanus* | VHL | PD-F1 | CGTTTAGTTTGGTTAGTCTTTTAGC |
|  |  | PD-R1 | ACTTTTATCTGTTTTGCTCTTCTCT |
|  | HIF1α-A | PD-F2 | ATCAGGCAGATGCGGCGAATG |
|  |  | PD-R2 | AAGCAAAAATCATCCCAGCGGTC |
|  | HIF1α-B | PD-F3 | TTTCTGTCCTCGGCGGCTTG |
|  |  | PD-R3 | AACGGTGTGGTGTGTGGAATAAAG |
|  | HIF2α-A | PD-F4 | TCAGTTTGGGGAGGTAGTGC |
|  |  | PD-R4 | TAAGAGTCGGCACAGAAATGA |
|  | HIF2α-B | PD-F5 | CGCCTTGTTTTTGTTATTGG |
|  |  | PD-R5 | GCAGTCAGTTTGGGTGTCAG |
