# Supplementary material for: Analysis of Multiplicity of Hypoxia-Inducible Factors in the Evolution of Triplophysa Fish (Osteichthyes: Nemacheilinae) Reveals Hypoxic Environments Adaptation to Tibetan Plateau
Source: Front Genet. 2020 May 12;11:433. doi: 10.3389/fgene.2020.00433 (PMC7235411; doi:10.3389/fgene.2020.00433)
Supplement: TABLE S2 — Gene specific primers with restriction enzyme sites for full-length pVHL and HIF-α isoforms. [file Table_2.DOCX]

**Tabel S2 Gene specific primers with restriction enzyme sites for full-length pVHL and HIF-α paralogs.**

| **Species** | **Gene** | **Primer name** | **Sequence** | **Restriction enzyme sites** |
| --- | --- | --- | --- | --- |
| *Triplophysa scleroptera* | VHL | F1 | CCGGAATTCGAATGCCTCAAGAGTTCCTGG | EcoRⅠ |
|  |  | R1 | TATCGCGGCCGCCTTATACGTTTTGCTCTTCTCTGT | NotⅠ |
|  | HIF1α-A | F2 | TAGCGAATTCGGATGGATGCAGGCGCAC | EcoRⅠ |
|  |  | R2 | CTAAGCGGCCGCTCACAGTGACAAAAAGCCG | NotⅠ |
|  | HIF1α-B | F3 | GCTAGTCGACCATGGATACTGGAGTTGTCAC | SalⅠ |
|  |  | R3 | TAGCGGTACCTCAGTTAACTTGGTCCAGAG | KPnⅠ |
|  | HIF2α-A | F4 | TAGCGAATTCGGATGACAGCCGAGAGAGA | EcoRⅠ |
|  |  | R4 | TGCATGGTACCCTACGTTGTCTGGTCCA | KPnⅠ |
|  | HIF2α-B | F5 | CCGGAATTCGAATGACAGTGGAAAAAGAGAAGAAGAGATG | EcoRⅠ |
|  |  | R5 | ATTTGCGGCCGCCTTAGGTGGCCTGATCTAATGCCTT | NotⅠ |
| *Paramisgurnus dabryanus* | VHL | F6 | TAGCGAATTCGGATGCCTCAAGAGCCTCAGGAA | EcoRⅠ |
|  |  | R6 | GGCTGGTACCTTATCTGTTTTGCTCTTCTCTGTTCTC | KPnⅠ |
|  | HIF1α-A | F7 | TAGCGAATTCGGATGGATACAGTCGCACCTGGGAA | EcoRⅠ |
|  |  | R7 | CTAAGCGGCCGCTCAGATAACTAAATCCAAGGCACACAG | NotⅠ |
|  | HIF1α-B | F8 | TAGCGAATTCGGATGGATACTGGAGTTGTCAC | EcoRⅠ |
|  |  | R8 | GGATGGTACCTCAATTAACTTGGTCCAGAG | KPnⅠ |
|  | HIF2α-A | F9 | TAGCGAATTCGGATGACAGCCGAGAAAGAG | EcoRⅠ |
|  |  | R9 | TTATGCGGCCGC CTACGTTGTCTGGTCGAG | NotⅠ |
|  | HIF2α-B | F10 | TAGCGAATTCGGATGACAGCAGAGAAGGAGAAGAAGA | EcoRⅠ |
|  |  | R10 | CTAAGCGGCCGCTTAGGTGGACTGATCTAATGCCTTT | NotⅠ |
