# Supplementary material for: Analysis of Multiplicity of Hypoxia-Inducible Factors in the Evolution of Triplophysa Fish (Osteichthyes: Nemacheilinae) Reveals Hypoxic Environments Adaptation to Tibetan Plateau
Source: Front Genet. 2020 May 12;11:433. doi: 10.3389/fgene.2020.00433 (PMC7235411; doi:10.3389/fgene.2020.00433)
Supplement: TABLE S3 — Gene information of T. scleroptera and P. dabryanus. [file Table_3.DOCX]

**Table S3 Genes information of *Triplophysa scleroptera* and *Paramisgurnus dabryanus.***

| **Latin name** | **Gene names** | **Length**  **(bp)** | **Amino acid**  **(AA)** | **Isoelectric point**  **(PI)** | **Molecular weight**  **(MW) (Da)** |
| --- | --- | --- | --- | --- | --- |
| *Triplophysa scleroptera* | HIF-1αA | 2058 | 686 | 5.30 | 76028.64 |
|  | HIF-1αB | 2328 | 776 | 5.20 | 85952.42 |
|  | HIF-2αA | 2529 | 843 | 5.98 | 94575.10 |
|  | HIF-2αB | 2451 | 817 | 6.39 | 91302.40 |
|  | pVHL | 513 | 171 | 6.74 | 19673.63 |
| *Paramisgurnus dabryanus* | HIF-1αA | 2190 | 730 | 5.32 | 81076.54 |
|  | HIF-1αB | 2316 | 772 | 5.19 | 85694.71 |
|  | HIF-2αA | 2508 | 836 | 6.36 | 95202.67 |
|  | HIF-2αB | 2466 | 822 | 7.04 | 91985.00 |
|  | pVHL | 513 | 171 | 9.04 | 19790.92 |
