# Supplementary material for: Analysis of Multiplicity of Hypoxia-Inducible Factors in the Evolution of Triplophysa Fish (Osteichthyes: Nemacheilinae) Reveals Hypoxic Environments Adaptation to Tibetan Plateau
Source: Front Genet. 2020 May 12;11:433. doi: 10.3389/fgene.2020.00433 (PMC7235411; doi:10.3389/fgene.2020.00433)
Supplement: TABLE S4 — Comparison amino acid sequence similarity of pVHL and HIF-α isoforms between T. scleroptera and other species. [file Table_4.DOCX]

**Table S4 Comparison amino acid sequence similarity of pVHL and HIF-α paralogs between *Triplophysa scleroptera* and other species.**

| **Genes** | **Identity with (%)** | | | | | |
| --- | --- | --- | --- | --- | --- | --- |
|  | ***Paramisgurnus dabryanus*** | ***Triplophysa siluroides*** | ***Triplophysa dalaica*** | ***Danio rerio*** | ***Mus musculus*** | ***Homo sapiens*** |
| pVHL | 87.65% | 91.81% | 94.12% | 74.86% | 47.57% | 38.81% |
| HIF-1αA | 74.25% | 94.16% | 98.10% | 66.21% | 40.26% | 40.69% |
| HIF-1αB | 82.11% | 93.08% | 97.29% | 80.97% | 55.18% | 56.24% |
| HIF-2αA | 79.74% | 89.55% | 92.13%% | 73.32% | 51.13% | 52.04% |
| HIF-2αB | 82.14% | 93.14% | 98.65% | 79.28% | 53.77% | 54.34% |
