# Supplementary material for: Analysis of Multiplicity of Hypoxia-Inducible Factors in the Evolution of Triplophysa Fish (Osteichthyes: Nemacheilinae) Reveals Hypoxic Environments Adaptation to Tibetan Plateau
Source: Front Genet. 2020 May 12;11:433. doi: 10.3389/fgene.2020.00433 (PMC7235411; doi:10.3389/fgene.2020.00433)
Supplement: TABLE S5 — Amino acid variations of pVHL gene in T. scleroptera. [file Table_5.DOCX]

**Table S5 Amino acid variations of pVHL gene *in Triplophysa scleroptera .***

| **Varint** | **PROVEAN score** | **Prediction (cutoff= -2.5)** |
| --- | --- | --- |
| A19V | -0.623 | Neutral |
| T61M | 2.723 | Neutral |
| A98V | -0.358 | Neutral |
| P92T | -2.743 | Deleterious |
| S109T | 1.250 | Neutral |
