# Supplementary material for: Analysis of Multiplicity of Hypoxia-Inducible Factors in the Evolution of Triplophysa Fish (Osteichthyes: Nemacheilinae) Reveals Hypoxic Environments Adaptation to Tibetan Plateau
Source: Front Genet. 2020 May 12;11:433. doi: 10.3389/fgene.2020.00433 (PMC7235411; doi:10.3389/fgene.2020.00433)
Supplement: TABLE S6 — List of HIF-α genes in representive invertebrates and vertebrates. The black filled pentagram and empty pentagram represent the existency and inexistence of HIF gene in the selected species. [file Table_6.DOCX]

**Table S6. List of HIF-α genes in representive invertebrates and vertebrates. The black filled pentagram and empty pentagram represent the existency and inexistence of HIF gene in the selected species.**

| **Lineage** | **Common name** | **HIF-1α** | **HIF-2α** | **HIF-3α** | **HIF-4α** |
| --- | --- | --- | --- | --- | --- |
| Mammals | Human | ★ | ★ | ★ | ☆ |
|  | Dog | ★ | ★ | ★ | ☆ |
|  | Mouse | ★ | ★ | ★ | ☆ |
|  | Rat | ★ | ★ | ★ | ☆ |
| Birds | Chicken | ★ | ★ | ☆ | ☆ |
|  | Duck | ★ | ★ | ☆ | ☆ |
|  | Zebra finch | ★ | ★ | ☆ | ☆ |
| Amphibian | Xenopus | ★ | ★ | ☆ | ☆ |
| Teleostei | Medaka | ★ | ★ | ☆ | ☆ |
|  | Tetraodon | ★ | ★ | ★ | ☆ |
|  | Amazon molly | ★ | ★ | ★ | ☆ |
|  | Chinese sucker | ★ | ★ | ★ | ☆ |
|  | Grouper | ★ | ☆ | ☆ | ★ |
|  | Fugu | ★ | ★ | ★ | ☆ |
|  | Grass carp | ★ | ★ | ☆ | ★ |
|  | Channel Catfish | ★ | ★ | ★ | ☆ |
|  | Triplophysa scleroptera | HIF-1αA/ HIF-1αB | HIF-2αA/ HIF-2αB | ☆ | ☆ |
|  | Paramisgurnus dabryanus | HIF-1αA/ HIF-1αB | HIF-2αA/ HIF-2αB | ☆ | ☆ |
|  | Triplophysa microps | HIF-1αA/ HIF-1αB | HIF-2αA/ HIF-2αB | ☆ | ☆ |
|  | Triplophysa siluroides | HIF-1αA/ HIF-1αB | HIF-2αA/ HIF-2αB | ☆ | ☆ |
|  | Zebrafish | HIF-1αA/ HIF-1αB | HIF-2αA/ HIF-2αB | ★ | ☆ |
| Holoste | Spotted gar | ★ | ★ | ★ | ☆ |
| Sarcopterygii | Coelacanth | ★ | ★ | ☆ | ☆ |
| Cephalochordata | Lancelet | HIFα | ☆ | ☆ | ☆ |
| Chromadorea | C. elegans | HIFα | ☆ | ☆ | ☆ |
