# Supplementary material for: Analysis of Multiplicity of Hypoxia-Inducible Factors in the Evolution of Triplophysa Fish (Osteichthyes: Nemacheilinae) Reveals Hypoxic Environments Adaptation to Tibetan Plateau
Source: Front Genet. 2020 May 12;11:433. doi: 10.3389/fgene.2020.00433 (PMC7235411; doi:10.3389/fgene.2020.00433)
Supplement: TABLE S7 — One-ratio model analysis of HIF-αs and pVHL gene. [file Table_7.DOCX]

**Table S7 One-ratio model analysis in 5 genes.**

| **Genes** | **Models** | **-lnL** | **2△(lnL)** | **P Value** | **ωValues** |
| --- | --- | --- | --- | --- | --- |
| HIF-1αA | M0(one-ratio) | 11712.885 |  |  | ω0=0.171 |
|  | M0(constrained) | 12390.426 | 1355.08 | p<0.001 | ω0=1 |
| HIF-1αB | M0(one-ratio) | 14108.660 |  | p<0.001 | ω0= 0.159 |
|  | M0(constrained) | 14977.936 | 1738.55 | p<0.001 | ω0=1 |
| HIF-2αA | M0(one-ratio) | 7746.019 |  | p<0.001 | ω0= 0.163 |
|  | M0(constrained) | 8214.640 | 937.24 | p<0.001 | ω0=1 |
| HIF-2αB | M0(one-ratio) | 8115.146 |  | p<0.001 | ω0= 0.161 |
|  | M0(constrained) | 8630.543 | 1030.79 | p<0.001 | ω0=1 |
| pVHL | M0(one-ratio) | 3829.088 |  | p<0.001 | ω0= 0.161 |
|  | M0(constrained) | 4083.401 | 508.62 | p<0.001 | ω0=1 |
