# Supplementary material for: Analysis of Multiplicity of Hypoxia-Inducible Factors in the Evolution of Triplophysa Fish (Osteichthyes: Nemacheilinae) Reveals Hypoxic Environments Adaptation to Tibetan Plateau
Source: Front Genet. 2020 May 12;11:433. doi: 10.3389/fgene.2020.00433 (PMC7235411; doi:10.3389/fgene.2020.00433)
Supplement: TABLE S8 — Selective pressure analyses of hypoxia-inducible factor and pVHL using PAML Branch-Site Model. [file Table_8.DOCX]

**Table S8**. **Selective Pressure Analyses of Hypoxia-inducible factor and pVHL using PAML Branch-Site Model.**

| **Genes** | **Branch Site Model** | **-lnL** | **2△(lnL)** | **P Value** | **ωValues** | **Positively Selected Sites** |
| --- | --- | --- | --- | --- | --- | --- |
| HIF-1αA | Branch (latest common ancestral branch of *Triplophysa*) |  |  |  |  |  |
|  | Null | 18057.167 |  |  | ω0=0.095, ω1=1.000  ω2a=1.000, ω2b=1.000 |  |
|  | Alternative | 18044.246 | 25.842 | 3.705e-07 | ω0=0.098, ω1=1.000  ω2a=101.941,ω2b=101.941 | 130 **V** 0.537, 340 **N** 0.989*,424**I** 0.784, 445 **R** 0.774  455 **C** 0.903, 579 **T** 0.873,640 **L** 0.577, 760 **G** 0.530  762 **S** 0.696, 763 **P** 0.891,765 **I** 0.629, 766 **P** 0.849  769 **H** 0.905, 770 **F** 0.869,772 **F** 0.893, 774 **F** 0.591 |
| HIF-1αB | Branch (latest common ancestral branch of *Triplophysa*) |  |  |  |  |  |
|  | Null | 17948.070 |  |  | ω0=0.092, ω1=1.000  ω2a=1.000, ω2b=1.000 |  |
|  | Alternative | 17948.070 | 0 | 1 | ω0=0.0916, ω1=1.000  ω2a=1.000, ω2b=1.000 |  |
| HIF-2αA | Branch (latest common ancestral branch of *Triplophysa*) |  |  |  |  |  |
|  | Null | 20114.580 |  |  | ω0=0.118, ω1=1.000  ω2a=1.000, ω2b=1.000 |  |
|  | Alternative | 20114.580 | 0 | 1 | ω0=0.118, ω1=1.000  ω2a=1.000, ω2b=1.000 |  |
| HIF-2αB | Branch (latest common ancestral branch of *Triplophysa*) |  |  |  |  |  |
|  | Null | 18491.675 |  |  | ω0=0.091, ω1=1.000  ω2a=1.000, ω2b=1.000 |  |
|  | Alternative | 18491.675 | 0 | 1 | ω0=0.091, ω1=1.000  ω2a=1.000, ω2b=1.000 |  |
| P-VHL | Branch (latest common ancestral branch of *Triplophysa*) |  |  |  |  |  |
|  | Null | 4345.207 |  |  | ω0= 0.134, ω1=1.000  ω2a=1.000, ω2b=1.000 |  |
|  | Alternative | 4345.207 | 0 | 1 | ω0= 0.134, ω1=1.000  ω2a=1.000, ω2b=1.000 |  |
|  | Branch (terminal branch of *Triplophysa scleroptera* ) |  |  |  |  |  |
|  | Null | 4343.993 |  |  | ω0=0.132, ω1=1.000  ω2a=1.000, ω2b=1.000 |  |
|  | Alternative | 4343.991 | 0.02 | 8.875e-01 | ω0= 0.132, ω1=1.000  ω2a=1.143,ω2b=1.143 | 22 **A** 0.649, 64 **T** 0.635,101 **A** 0.631, 112 **S** 0.671 |

**(** Note: * mean pp > 95%**)**
